# A processive phosphorylation circuit with multiple kinase inputs and mutually diversional routes controls G1/S decision

**DOI:** 10.1038/s41467-020-15685-z

**Published:** 2020-04-15

**Authors:** Rainis Venta, Ervin Valk, Mihkel Örd, Oleg Košik, Kaur Pääbo, Artemi Maljavin, Rait Kivi, Ilona Faustova, Nastassia Shtaida, Martin Lepiku, Kaidi Möll, Andreas Doncic, Mardo Kõivomägi, Mart Loog

**Affiliations:** 1 Institute of Technology, University of Tartu, Tartu, 50411 Estonia; 2 Department of Biology, Stanford University, Stanford, CA 94305 USA

**Keywords:** Cell division, Cell signalling

## Abstract

Studies on multisite phosphorylation networks of cyclin-dependent kinase (CDK) targets have opened a new level of signaling complexity by revealing signal processing routes encoded into disordered proteins. A model target, the CDK inhibitor Sic1, contains linear phosphorylation motifs, docking sites, and phosphodegrons to empower an N-to-C terminally directed phosphorylation process. Here, we uncover a signal processing mechanism involving multi-step competition between mutually diversional phosphorylation routes within the S-CDK-Sic1 inhibitory complex. Intracomplex phosphorylation plays a direct role in controlling Sic1 degradation, and provides a mechanism to sequentially integrate both the G1- and S-CDK activities while keeping S-CDK inhibited towards other targets. The competing phosphorylation routes prevent premature Sic1 degradation and demonstrate how integration of MAPK from the pheromone pathway allows one to tune the competition of alternative phosphorylation paths. The mutually diversional phosphorylation circuits may be a general way for processing multiple kinase signals to coordinate cellular decisions in eukaryotes.

## Abstract (web summary)

The decision of whether and when a cell divides is tightly controlled. Here, the authors show in yeast that there is a multi-step competition between different phosphorylation states and sites in the S phase CDK-Sic1 complex, which controls Sic1 degradation and coordinates the precise timing of the G1/S transition.

## Introduction

Recent works on multisite phosphorylation networks of cyclin-dependent kinase (CDK) have revealed a new level of signal processing complexity that is based on signaling routes encoded into disordered proteins or disordered regions of proteins^1–4^. As the master regulators of the cell division cycle, CDKs use a combination of different docking interactions and activity thresholds to temporally order the phosphorylation of hundreds of their targets in the cell cycle^5–13^. Most of the CDK targets are phosphorylated at multiple sites distributed along disordered regions within these proteins^14^. In G1 phase, Cyclin-dependent Kinase Inhibitors (CKIs), like disordered protein Sic1 (Substrate Inhibitor of Cdk)^15,16^ in yeast, or p27 in humans^17^, ensure that S- and M-phase promoting CDK activity is suppressed to allow origin licensing and other G1 processes that require low CDK activity^16,18^. Interestingly, both Sic1 and p27 have the seemingly controversial functions of acting simultaneously as an inhibitor of and a substrate for CDK. Although its inhibitory function keeps CDK activity low via tight nanomolar affinity, the substrate function involves phosphorylation-induced degradation of CKI via the ubiquitin–proteasome pathway at G1/S, to unleash free S-phase CDK activity^19,20^.

**Fig. 1:**
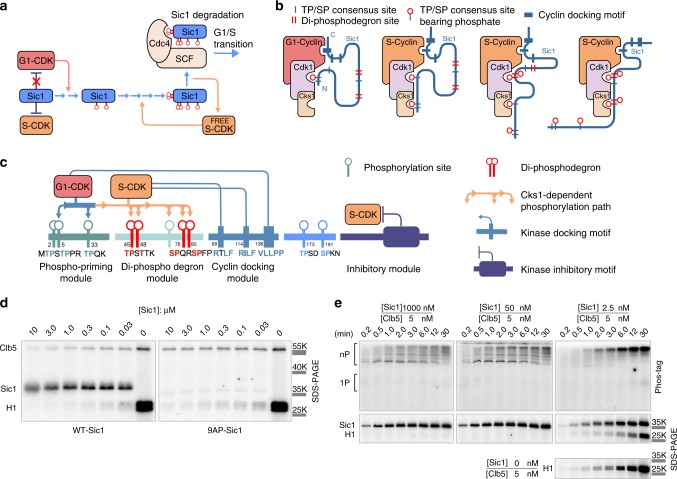
Processive phosphorylation of Sic1 within the S-CDK–Sic1 inhibitory complex. **a** A model for G1/S transition switch in *S. cerevisiae*. The S-CDK inhibitor Sic1 first undergoes priming phosphorylation by G1-CDK, followed by phosphorylation of the degrons by a small fraction of uninhibited S-CDK, directing Sic1 to Cdc4-SCF-mediated ubiquitination and proteolysis. Further release of free S-CDK accelerates Sic1 degradation. **b** The general dynamics of sequential phosphorylation of Sic1 by cyclin–Cdk1–Cks1 complexes. The disordered N-terminal non-inhibitory fragment (amino acids 1–215) is shown to take different configuration when binding to the CDK complexes to facilitate N-to-C terminally directed multisite phosphorylation. **c** A scheme showing key interactions, modules, and sequence motifs involved in Sic1 regulation. **d** An autoradiograph of a ^32^P kinase assay showing the intracomplex phosphorylation of Sic1 within reconstituted S-CDK–Sic1 complex (Clb5–Cdk1–Cks1–Sic1) at different Sic1 concentrations. Histone H1 was used as an external substrate. A Sic1 version with all CDK sites mutated to alanines was included as a control (right panel). **e** Kinase assays with the excess of Sic1 concentration over the S-CDK complex, or the excess of S-CDK concentration over Sic1, were followed in Phos-tag and conventional (lower panels) SDS–PAGE. The corresponding autoradiographs are shown. In case of the control without Sic1, no ^32^P signal was observed in Phos-tag gel. The experiments for **d** and **e** were performed twice with similar result. The full gel radiographs are presented in Supplementary Fig. 7. Source data are provided as a Source Data file.

In such a mutually antagonistic relationship, Sic1, an intrinsically disordered protein with 10 CDK phosphorylation sites, inhibits the S-phase CDK complex in budding yeast, whereas the same S-CDK targets it for degradation by multisite phosphorylation of the Sic1 N terminus at G1/S transition (Fig. 1a). Degradation of phosphorylated Sic1^19,21^ causes a release of free S-CDK, which, in turn, further accelerates the phosphorylation and degradation of Sic1 by positive feedback^1,16,22–25^. The resulting burst of free S-CDK triggers the S phase. The first steps of the Sic1 multisite phosphorylation process are primed by G1-CDK, a complex that is not inhibited by Sic1^1,26,27^. The phosphates are added stepwise via docking by phospho-adaptor Cks1 in the N-to-C-terminal direction with the help of a complex network of interactions (Fig. 1b, c). The free emerging S-CDK will finalize the cascade by completing the phosphorylation of the two di-phosphodegrons required for degradation.

It has remained an enigma, however, how is it possible that S-CDK is inhibited by Sic1, but also acts as a major driving force for Sic1 phosphorylation and degradation at G1/S transition. This double-negative feedback loop also assumes controversial simultaneous roles for Sic1 as a phospho-degradable substrate and an inhibitor, which would require a barrier preventing Sic1 to be phosphorylated prematurely by leaking kinase activity within the inhibitory complex, or otherwise, tight control of G1/S would be lost.

In the current study, we show that within the inhibitory complex, while being inhibited toward other targets, the S-CDK complex (Clb5–Cdk1–Cks1) still efficiently phosphorylates Sic1. We found that this intracomplex multisite phosphorylation network contains two competing pathways, one leading to degron phosphorylation, and another that functions as a diversionary path that terminates the degron route. Using fluorescent live-cell microscopy, we demonstrate how diversionary phosphorylation by S-CDK within the inhibitory complex prevents the kinase signal leakage via the phospho-degron route. We also show that the diversionary route can be primed by MAPK from the pheromone pathway to ensure G1 arrest, or alternatively, the diversion can be outcompeted by priming phosphorylations within the phospho-degron route by the G1-CDK complex, which accumulates after the Start decision. This paper will lay out a mechanism that enables the disordered Sic1 molecule, with its linearily encoded phosphorylation pattern, to act as a processor that integrates multiple kinase inputs.

## Results

### Processive phosphorylation within the inhibitory complex

To study the phosphorylation leakage within the Sic1–Clb5–Cdk1–Cks1 inhibitory complex, we reconstituted the complex in vitro, and performed a standard kinase assay followed by SDS–PAGE ^32^P autoradiography. We observed that within a reconstituted complex, while being inhibited toward a control target histone H1, the S-CDK complex (Clb5–Cdk1–Cks1) still efficiently phosphorylates Sic1 (Fig. 1d). Using a Phos-tag reagent that provides better resolution of phosphorylated forms in the gels^28^, we observed an initial very fast multisite phosphorylation stage followed by termination (Fig. 1e; Supplementary Fig. 1a). The intensity and the overall pattern of phosphorylation were found to be independent of the fold-excess of Sic1 over the Clb5–Cdk1–Cks1 (CDK complex). Contrarily, an assay mixture with an excess of CDK over Sic1 yielded a full unterminated multisite phosphorylation pattern, apparently due to extra-complex targeting of Sic1 in the inhibitory complex by uninhibited CDK (Fig. 1e). We also performed a similar experiment as in Fig. 1e, except that the inhibitory complex was pre-formed using an excess of Sic1 version with alanine-mutated N-terminal CDK motifs (8AP-Sic1). Next, wild-type full-length Sic1 or non-inhibitory Sic1ΔC was added (Supplementary Fig. 1b). No considerable phosphorylation of added Sic1 was observed, which clearly indicates that the phosphorylation occurs only within the complex and no exogeneous substrate is targeted.

Qualitative mass spectrometry analysis of the terminated inhibitory complex revealed peptides containing phosphorylated CDK sites T5, T45, and T173 (Supplementary Fig. 1c). The site T5 is a priming site of the N-to-C-terminal Cks1-dependent degron path^1,2^ (Fig. 1b, c). The site T173, however, does not yet have a well-explained mechanistic function, but has been proposed to either interfere with Cdc4-SCF binding or contribute to S-CDK inhibition^29–32^. We hypothesized that the site T173, bearing an ideal Cks1-binding phospho-motif^33^, and located about a hundred amino acids downstream from the N-terminal dergon sites, could serve as a diversional Cks1-binding site that could cause the intracomplex termination of the fast processive phosphorylation of N-terminal pathway.

**Fig. 2:**
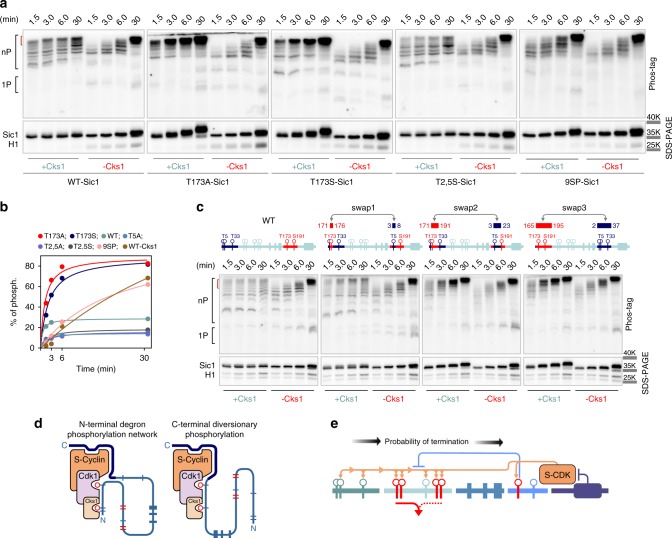
The intracomplex phosphorylation of N-terminal multisite phosphorylation network of Sic1. **a** The intracomplex phosphorylation of different Sic1 phosphorylation site mutants by Clb5–Cdk1 were followed in the presence or absence of the phospho-adaptor protein Cks1. The 9SP-Sic1 denotes a mutated form where all TP phosphorylation sites were mutated to SP. The upper gels contained Phos-tag to resolve phosphorylated forms. The control incubations shown in the lower panels are ordinary SDS–PAGE and also contain the external substrate Histone H1. **b** Quantified time courses of the hyperphosphorylated forms from the assays shown in **a**. The quantified segment is indicated by red bracket on the left in the **a**. The 100% phosphorylation corresponds to total radioactivity quantified from all phospho-bands of the corresponding lane. **c** Three different versions of Sic1 with swapped regions around Cdk1 sites T5 and T173, as indicated on the schemes, were analyzed using Phos-tag kinase assay as in **a**. The experiments for **a** and **c** were performed twice with similar result. **d** Two alternative and mutually competitive phosphorylation modes of Sic1 within the inhibitory complex. S-CDK phosphorylates Sic1 within the inhibitory complex, targeting both N- and C-terminal sites. In case phosphorylated T173 binds to the Cks1 pocket, the N-terminal phosphorylation process is terminated. **e** Schematic depiction of intracomplex phosphorylation steps and increasing probability of their termination by diversionary phosphorylation. The full gel radiographs are presented in Supplementary Fig. 7. Source data are provided as a Source Data file.

Both the T173S and T173A mutations prompted rapid accumulation of hyperphosphorylated forms (Fig. 2a, b), which is in agreement with the hypothesis as the phospho-binding pocket of Cks1 binds only phospho-threonine containing CDK sites and not phospho-serines^2,33^. A similar result was obtained when Cks1 was omitted from the reaction (Supplementary Fig. 1a). Contrarily, phosphorylation of a non-inhibitory C-terminally truncated version of Sic1ΔC^34^ did not show any sensitivity to T173 mutation (Supplementary Fig. 1d). Finally, it is important to note that the T173A or T173S mutations had no effect on the ability of Sic1 to inhibit H1 phosphorylation, arguing against the role of T173 in inhibitory interaction^31^ (Fig. 2a).

In contrast, when the N-terminal priming site T5 was mutated either to serine or alanine, relatively less hyperphosphorylated forms, compared to the wild-type Sic1, were observed (Fig. 2a, b; Supplementary Fig. 1e). Furthermore, when all the threonine-based CDK sites were mutated to serines (9SP-Sic1), the intracomplex phosphorylation ladder resembled the experiment lacking Cks1 (Fig. 2a, b). Interestingly, the S-CDK-specific RXL docking motifs^35–38^ did not influence the intracomplex process, whereas a mutation in the inhibitory domain known to reduce the inhibitory potency of Sic1^34^ increased the overall phosphorylation (Supplementary Fig. 1e). Similarly, the mitotic B-type cyclin complexes Clb3– and Clb2–Cdk1–Cks1 exhibited similar patterns of intracomplex phosphorylation, suggesting its role also in mitotic exit when Sic1 is starting to accumulate (Supplementary Fig. 1f). These results suggest that intracomplex phosphorylation of N-terminal degron route is supported by the Cks1 docking mechanism and the inhibitory domain interaction.

To answer the question why N-terminal T5 can act as a Cks1-binding primer site that enhances multisite phosphorylation, but the potential diversionary site T173 acts via Cks1 as a dead-end terminator, we swapped three different sequence segments surrounding sites T5 and T173, and measured the intracomplex phosphorylation in vitro using the Phos-tag assay (Fig. 2c). Swapping the minimal CDK (swap 1) motif only mildly affected the termination, and the dynamics of the phosphorylation ladder. In contrast, when the swapped segment contained longer sequences extending toward the C-terminal direction from T5 or T173 (swap 2, 3), no termination was observed (Fig. 2c). These data suggest that the sequence segment at the C-terminal side from the T173 CDK consensus site contains a motif that apparently adds affinity for pT173–Cks1 interaction, possibly via binding to the surface of the Cks1–Cdk1 module. Furthermore, the amino acid sequence surrounding the T173 position is highly conserved even among distant yeast species (Supplementary Note 1). This finding is surprising and demonstrates that different pTP sites can have considerably different affinity for Cks1 that depend on the wider sequence context along the disordered protein segment. It allows the pTP sites to act either as enhancers or inhibitors of the multisite phosphorylation process, and it explains the reason behind the observed diversionary intracomplex termination pattern.

Finally, to demonstrate that Cks1 was bound to the inhibitory complex via the site pT173 in vivo, we performed a co-immunoprecipitation of the Clb5–TAP–Cdk1–Cks1–Sic1–6HA complex via the TAP-tag from two strains with either wild-type SIC1–6HA or T173A–SIC1–6HA alleles. Cks1 was co-immunoprecipitated from a strain expressing the wild-type Sic1–6HA protein, but not T173A–Sic1–6HA (Supplementary Fig. 1g), which confirms that T173 is phosphorylated and the terminated complex is formed in vivo. The presented experiments support the model of two alternative phosphorylation routes competing for the Cks1 phospho-pocket and for the active site of CDK within the inhibitory complex, with the T173 primed route leading to termination of phosphorylation of the N-terminal degron route (Fig. 2d, e).

### Phosphorylation site-specificity is lost within the complex

**Fig. 3:**
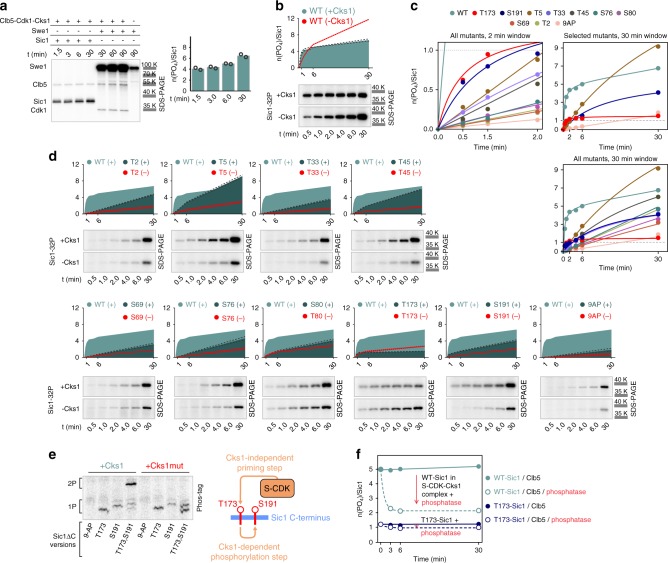
The phosphorylation site-specificity is lost within the inhibitory complex. **a** The stoichiometry of intracomplex phosphorylation of Sic1 in vitro was determined by using inhibitory tyrosine kinase Swe1. Mono-phosphorylation of Y19-Cdk1 by Swe1 was used as a quantitative reference to estimate the number of phosphates added per molecule of Sic1 in a separate S-CDK (Clb5–Cdk1–Cks1) kinase assay loaded on the same gel. Bars represent the mean level derived from the data of two values that are obtained from two independent experiments. The values are plotted individually by the open circles. **b** The stoichiometry for intracomplex phosphorylation of Sic1 in the presence or absence of Cks1 in the assay mixture. The dotted lines represent standard deviation of the time-course obtained from two independent experiments. **c** Plots demonstrating the Clb5–Cdk1–Cks1–Sic1 intracomplex phosphorylation dynamics of Sic1 versions containing only selected CDK sites with the rest of the CDK sites mutated to alanines. The plots obtained from two independent experiments were nearly overlapping. **d** The individual phosphorylation profiles shown on **c** plotted together with the wild-type Sic1 phosphorylation profile for comparison. The 9AP denotes the version with all CDK sites mutated to alanines. The dotted lines represent standard deviation of the time-course obtained from two independent experiments. The full Sic1 amino acid sequences can be found in Source data file. **e** In vitro phosphorylation of Sic1ΔC versions containing the indicated CDK sites with the rest of the CDK sites mutated to alanines to demonstrate the Cks1-mediated double-phosphorylation of diversionary sites using Clb5–Cdk1–Cks1 complex. Autoradiographs of Phos-tag SDS–PAGE are shown. **f** Plot demonstrating the dephosphorylation of Sic1 within Sic1–Clb5–Cdk1–Cks1 complex by lambda phosphatase. The plotted trendlines were obtained by fitting the data points as described in the Methods section. The plots obtained from two independent experiments were overlapping. The experiments for **a**–**f** were performed twice with similar result. The full gel radiographs are presented in Supplementary Fig. 7. Source data are provided as a Source Data file.

We estimated the stoichiometry of the intracomplex phosphorylation of Sic1 relative to the inhibited CDK complex using Cdk1 active-site titration with the inhibitory tyrosine kinase Swe1 (Fig. 3a, b). It was revealed that in case of wild-type Cks1 up to 6 phosphates were added to Sic1 per CDK complex, while in case of reaction without Cks1, strikingly, the number of phosphates per Sic1 largely exceeded the number of CDK consensus sites (Fig. 3b). This result suggests that in case of no pT173–Cks1-mediated termination, S-CDK continues to phosphorylate different sites in the inhibitory complex. As the number of phosphates added surpasses the number of CDK consensus sites in Sic1, the non-consensus sites must also be targeted. This suggests that in the tight inhibitory complex, CDK site-specificity is lost.

Interestingly, when Sic1 versions containing only individual single sites were tested in the inhibitory complex assay, we observed that compared to the relatively slow N-terminal phosphorylation sites, both sites T173 and S191 were phosphorylated rapidly almost up to 1 phosphate per Sic1 molecule (Fig. 3c). In case of T173, the process was subsequently terminated, while the S191 version continued accumulating phosphates, suggesting that pT173 locks the process via Cks1-dependent dead-end termination mode. Strikingly, all Sic1 versions that contain a single CDK site, except the construct with only T173, displayed a linear increase of the phosphorylation signal that surpassed 1:1 stoichiometry per inhibitory complex (Fig. 3c, d). The Sic1 version with the T5 site alone was able to receive up to 9 phosphates despite being the only CDK consensus site in the molecule. This suggests that the pT5–Cks1 interaction combined with the inhibitory docking via the C-terminal domain of Sic1 creates a powerful induced proximity between the kinase and its substrate, and the consensus site-specificity ceases to be important. This, however, would present a huge risk for signal leak—a design paradox of the double-negative feedback-based kinase-inhibitor switches, discussed in the introductory paragraph. Also, site T45, as in T5, bears an ideal consensus motif for Cks1-binding, PxTP^2,33^, which accumulates more than four phosphates per inhibited CDK via the intracomplex route. Although the phosphorylation stoichiometry of the other single-site constructs was lower, they all, including 9AP-Sic1, accumulated more than one phosphate per complex, which suggests that random serines and threonines get phosphorylated. We also demonstrated that in the presence of Cks1 higher stoichiometry was achieved in all constructs except T173–Sic1, which suggests that in addition to the inhibitory domain interaction, Cks1 contributes to the phosphorylation of non-consensus sites (Fig. 3d).

In addition, the sites T173 and S191 are at an optimal position from each other for a Cks1-mediated phosphorylation step. Indeed, in a Phos-tag kinase assay, a construct that only contained sites T173 and S191 rapidly accumulated a double-phosphorylated form in a Cks1 phospho-pocket dependent manner (Fig. 3e). In addition, externally added lambda phosphatase can remove all, except two, phosphates added via the intracomplex process in case of wild-type Sic1 (Fig. 3f). However, in case of the single CDK site version with T173 (T173–Sic1), the phosphatase has no effect, which suggests that the pT173-driven termination is highly irreversible. It is important to note that the terminated complex is different from the inhibitory complex, as both are inhibited toward the external substrates, but only the terminated complex is inhibited toward the N-terminal degron network.

These data further supported our hypothesis that the diversionary T173–S191 pathway may be used as a lock to prevent the premature N-terminal signal leak via highly efficient phosphorylation of N-terminal sites. The system may even act as a rheostat, integrating opposite kinase signaling pathways into T173 to tune and control the leaking CDK thresholds for preventing premature G1/S transition. This idea is supported by the fact that the site T173 has been reported to be a target of stress response MAPKs Hog1 and Mpk1 that delay the G1/S transition^29,31,32^.

### MAPK Fus3 phosphorylates T173 in vivo and in vitro

**Fig. 4:**
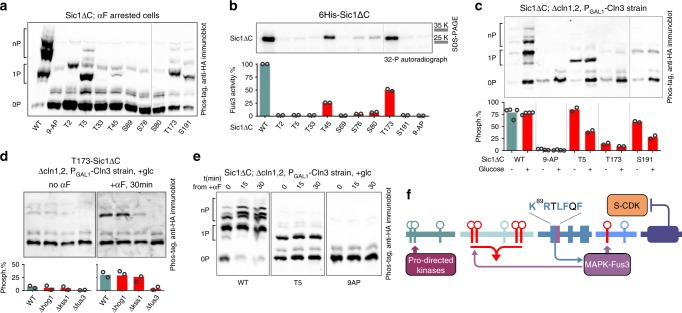
Analysis of the phosphorylation pattern of Sic1 in G1-arrested cells. **a** A Phos-tag western blotting analysis of the phosphorylation of Sic1ΔC-3HA versions containing only the indicated CDK consensus sites, with all other CDK sites mutated to alanines. All western blots in this figure were performed using the 3HA-tagged Sic1ΔC versions expressed under ADH1 promoter. **b** In vitro kinase assay showing phosphorylation of Sic1 versions with single phosphorylation sites by MAPK Fus3. An autoradiograph of an ordinary SDS–PAGE is shown. Bar diagrams in this figure represent the mean level derived from the data of two values that are obtained from two independent experiments. The values are plotted individually by the open circles. **c** The phosphorylation shifts of Sic1ΔC versions carrying only the phosphorylation sites T5, T173, or S191 visualized by Phos-tag western blotting of the cell extracts obtained from G1-arrested cultures. The cells were arrested in G1 by suppression of GAL-CLN3 expression by addition of glucose in a Δcln1 Δcln2 strain background. The Sic1ΔC version with all nine CDK consensus phosphorylation sites mutated to alanines was used as a control. The shift values were calculated as percentage of quantified phosphorylated bands from the sum of non-phosphorylated and phosphorylated bands. **d** The effect of the pheromone on T173 phosphorylation shift in cells arrested in G1 as in **c**. Strains expressing the T173–Sic1ΔC in wild-type and MAPKs FUS3, HOG1, and KSS1 deletion backgrounds were compared. Mean values presented in the chart were calculated as in **c**, and are obtained from two independent experiments. **e** The G1 arrest was induced as in **c** and **d**, then α-factor was added to the cultures and timepoints collected after indicated time The wild-type Sic1ΔC, T5-Sic1ΔC, and 9AP-Sic1ΔC strains were used. **f** A diagram showing MAPK Fus3 input phosphorylation sites, MAPK docking motif KRXXFXF overlapping with the S-CDK docking motif RXLF, and T5 as the input site for generic proline-directed kinase signal. The experiments for **a**–**e** were performed twice with similar result. The full gel radiographs and blots are presented in Supplementary Fig. 7. Source data are provided as a Source Data file.

The presence of pheromone in the environment leads to a period of elongated G1, where the cell needs to decide between two opposing cell faiths: cell cycle or mating. To see whether the pheromone signal shifted the balance of Sic1 degron and diversionary pathway, we studied the phosphorylation of Sic1 in response to pheromone. Using Phos-tag SDS–PAGE and western blotting of the extracts from cells arrested in G1 by α-factor, we observed a pattern of three phosphorylation bands using both full-length Sic1 and non-inhibitory Sic1ΔC (Supplementary Fig. 2a). Next, by analyzing the phosphorylation bands in the single-site Sic1ΔC constructs, it was possible to extrapolate that the pattern of the mobility shifts was mostly composed of phosphorylations at positions T5, T173, and less at S191 and T45 (Fig. 4a; Supplementary Fig. 2a). Also, the phosphorylation shifts were not dependent on Cln3, the only cell cycle-related cyclin present in G1^39,40^ (Supplementary Fig. 2b). However, the kinase active during the arrest, the final output signal of the pheromone pathway, MAPK Fus3, phosphorylated Sic1 efficiently in vitro at site T173, and to a lesser extent at site T45 (Fig. 4b). To validate that T173 is a Fus3 target in vivo, we used a strain background where, instead of the α-factor, G1 arrest was enforced by suppressing the expression of G1 cyclins. After arresting the cells in G1, only a minor T173 shift was observed (Fig. 4c, d). Addition of the pheromone induced the pT173 shift, suggesting that Fus3 is the kinase that phosphorylates T173 (Fig. 4d). No T173 shift was observed after deleting *FUS3*, while *HOG1* deletion had no effect, and deletion of *KSS1*, a MAPK partially redundant with Fus3^41^, caused an intermediate shift (Fig. 4d). These data suggest that the pheromone pathway may enhance the diversional termination of the Sic1 degron pathway phosphorylation as an additional mechanism to support Far1 enforced G1 arrest. Secondly, by analyzing the phosphorylation dynamics of the priming site T5 of the N-terminal pathway, we observed that after the Cdk1-dependent phosphorylation during the S and M phases was subtracted via specific Cdk1 inhibition, a constant phosphorylated/non-phosphorylated ratio of roughly 1:1 remained throughout the cell cycle (Supplementary Fig. 2c). The shift of T5 was unchanged during G1 arrest regardless of pheromone pathway activation (Fig. 4e). It is possible that this constant phosphorylation status could be caused by a sum of different proline-directed kinases, because we were not able to single out any individual one responsible from a large set of candidate kinase deletions (Supplementary Fig. 2d). It is possible that keeping a constant 50% phosphorylation equilibrium against the phosphatase would offer a good buffer against fluctuations of the activities of any individual proline-directed kinases in G1, and thereby, protect against priming for Sic1 degradation by erroneous signals. Taken together, both priming sites of the alternative pathways, T5 and T173, are phosphorylated during pheromone arrest (Fig. 4f). This suggests that the emerging S-CDK complex will meet a fixed ratio of initial pathway inputs. This ratio, however, could be shifted when G1-CDK accumulates and the pheromone signal fades at G1/S.

In order to directly test whether the pheromone pathway acts via T173 in Sic1, we performed a classical halo assay to test the pheromone sensitivity by immersing paper discs into α-factor solution and placing the discs on agar plates to create a radially diffusing gradient of the pheromone (Supplementary Fig. 3a). In this way, the pheromone arrest can be evaluated by visually examining the growth of cells around the discs. We found that T173 was required for α-factor-induced arrest in the context of overexpressed S-phase cyclin Clb5, thus suggesting that Fus3 directly targets T173 to induce intracomplex termination of Sic1 degron phosphorylation by S-CDK.

### Diversional pathway prevents the leakage of CDK activity

**Fig. 5:**
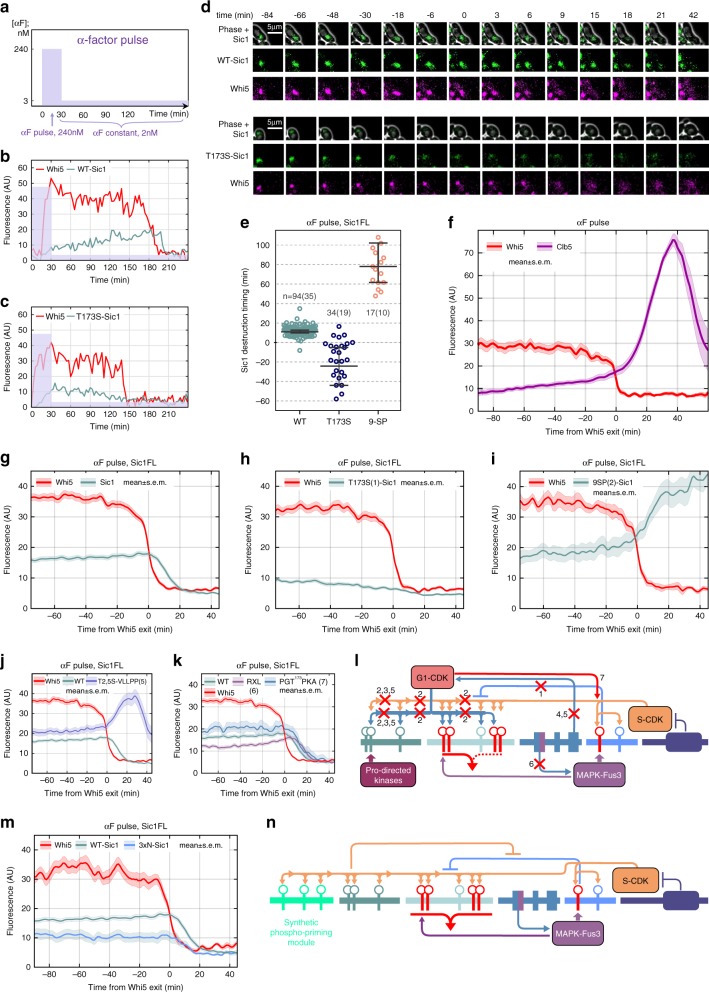
Single-cell analysis of Sic1 degradation using strains expressing Whi5-mCherry and Sic1-EGFP. **a** A scheme showing the profile of pheromone concentration applied during the microfluidics α-factor pulse experiments, designed to study Sic1 dynamics during the prolonged borderline arrest. **b**, **c** Examples of quantified nuclear Sic1-EGFP and T173S-Sic1-EGFP fluorescence signals in cells, which received the high α-factor pulse in G1. **d** Examples of time-lapse microscopy images around Start, which occurs at timepoint 0. The sample cells were chosen from 94 or 34 cells from WT-Sic1 and T173S-Sic1 strains, respectively. **e** Plots showing the distributions of Sic1 destruction timing values of individual cells for the indicated strains counted from 50% of Whi5 nuclear exit. The black lines denote the median value with 95% confidence intervals. The number of cells (*X*) observed over a number of colonies (*Y*) is given in form *n* = *X*(*Y*). 8 cells from T173S are out of the bounds of this plot. **f** Mean fluorescence levels for the time courses of Whi5-mCherry and Clb5-yeCitrine during the pulse experiment obtained by pooling together the fluorescence profiles of individual cells. The shaded areas around the mean profile here and in panels below represent ±s.e.m. **g**–**i** Mean fluorescence levels for strains with Whi5-mCherry and different Sic1-EGFP mutated versions. For the meaning of the numbers given in brackets see the **I** below. **j**, **k** Comparison of the mean fluorescence levels of Sic1-EGFP in indicated strains from the pulse experiment. In **j**, **k** Whi5-mCherry is shown for the strain with the wild-type Sic1 only. **l** A scheme explaining the interactions disconnected or created by the mutations in Sic1. The number above the cross marks or the red arrow indicate the strain number in **g**–**k** of this figure and in Supplementary Fig. 4g. For comprehensive explanations, please see Supplementary Note 2. **m** Mean fluorescence levels for Sic1 constructs with added N-terminal phosphorylation modules in an α-factor pulse experiment. Whi5-mCherry is shown for the strain with the wild-type Sic1 only. **n** A scheme explaining the effect of added N-terminal phosphorylation modules. Source data is available online, additional statistical information is provided in Supplementary Table 1.

To study the functional role of the diversional mechanism in Sic1 degradation, we set up a live-cell microscopy assay in a microfluidics device with strains expressing Whi5-*mCherry* and mutated versions of Sic1-EGFP^42^. As a reference point for Start, we used the timepoint at 50% nuclear exit of Whi5-*mCherry*, as described previously^43^ (Supplementary Fig. 3b). To measure Sic1 levels and degradation in the presence of the pheromone signal, we used a recently well-characterized regime with an initial short pulse of a high concentration of pheromone followed by constant low levels of pheromone^44,45^ (Fig. 5a). The fluorescence signals were quantified for cells that met the high pheromone pulse during G1. Such experiments have been reported to be particularly useful to dissect borderline arrest situations that possibly occur in the natural environment, when the cell-fate decision—either to divide or stay in G1 and mate—is not clearly pre-determined and the decision-making requires a longer time and presumably more information^44,45^. In such a borderline state at low pheromone levels, multiple antagonistic kinase inputs—G1-CDK & S-CDK, vs MAPK Fus3—are simultaneously present, and Sic1-EGFP levels can be analyzed both during a prolonged G1 arrest, and at G1/S transition. We also performed control experiments to ensure that the EGFP tag used does not affect the interaction of Sic1 with CDK complexes (Supplementary Fig. 3c, d).

During G1 arrest at low pheromone levels, the level of wild-type Sic1 increased gradually, yet strikingly, the T173S mutation led to a slow decline of Sic1 during the arrest. The wild-type Sic1 was degraded rapidly after Start, whereas the T173S mutation shifted the timing of the 50% fraction of Sic1 degradation about 40 min earlier compared to the wild-type Sic1, and it took place before Start in the majority of cells. Also, the degradation observed during the arrest was considerably slower compared to the wild-type Sic1 at G1/S (Fig. 5b–e, g, h; Supplementary Fig. 4i, j; for T173A-Sic1, see Supplementary Fig. 3e and Supplementary Table 1). Mutation of threonines to serines in N-terminal CDK sites reintroduced the wild-type-like slow accumulation pattern of Sic1 during prolonged borderline arrest, despite the presence of the T173S mutation (Fig. 5e, i). This indicates that the decline of the T173S-Sic1 levels was likely due to the inability of the inhibited S-CDK complex that accumulated during the arrest (Fig. 5f) to terminate the intracomplex phosphorylation of the N-terminal degron route, mediated by Cks1-pTP docking interactions. We did not observe any considerable difference in the dynamics of the wild-type, or T173S, versions of non-inhibitory Sic1ΔC-EGFP, which further supports the intracomplex mechanism (Supplementary Fig. 3f). Also, the VLLPP and T2,5S-VLLPP mutations increased Sic1 levels, which imply that accumulating G1-CDK also contributes to the adjustment of Sic1 levels during the borderline arrest (Fig. 5j, l, Supplementary Figs. 3g, 4g). Unexpectedly, the mutation in S-CDK specific RXL motifs (AXA2,3) suppressed Sic1 levels, although it did not show a considerable effect on the intracomplex process in vitro (Fig. 5k, Supplementary Fig. 1e). The ^89^RXL motif overlaps with the predicted MAPK docking site **KR**TL**F**Q**F**, which hints that this mutant was defective in Fus3-dependent phosphorylation of T173. We purified the wild-type and ^89^RXL-mutated Sic1 constructs with single Fus3-sites, T173–Sic1, and T45–Sic1, and tested their phosphorylation by Fus3 in vitro (Supplementary Fig. 3h). The rate of T173 phosphorylation was severely reduced in ^89^RXL-mutated form compared to T45, which suggests that an altered balance between the degron and diversion pathways may cause the lower Sic1 levels.

To further analyze the concept of a balance between the two alternative pathways, we introduced a G1-CDK specific consensus elements, a lysine in position +2 from T173 and proline in position −2 (T173-2P+2KA, the final sequence PGT^173^PKA)^46^ (Fig. 5k). As predicted, the levels of this mutant increased, presumably due to larger fraction of primed T173. Because Sic1 is not degraded during high pheromone arrest (Supplementary Fig. 3i), we can conclude that Fus3 alone is not able to fully phosphorylate the N-terminal degron network and cause the degradation. Thus, accumulating G1- and S-CDK activities are responsible for degrading Sic1 during the borderline arrest prior Whi5 nuclear exit. However, partially due to the intracomplex phosphorylation by S-CDK and partially due to the Fus3 input, the pT173 terminates the N-terminal route at early steps, and Sic1 is kept from declining before sufficient levels of G1- and S-CDK have accumulated. Interrupting the termination of intracomplex phosphorylation by T173S mutation results in strongly decreased Sic1 levels in G1, while directing G1-CDK input to T173 (T173-2P+2KA) or decreasing Fus3 input to T173 (RXL) either increases or decreases Sic1 stability, respectively (Fig. 5l). Conversely, decreasing the priming phosphorylation in the N-terminal sites by mutation of T5 or VLLPP docking site, results in increased Sic1 levels (Fig. 5l), indicating that priming phosphorylation of either the degron or the diversionary paths affects the stability of Sic1 in G1.

To explore whether it is possible to outcompete the diversional route by adding phosphorylation modules to the N terminus of Sic1, and thereby tune the Sic1 levels during the arrest, we introduced three optimal CDK sites, at Cks1 docking distances^2^ to the N terminus (Fig. 5m, n, Supplementary Fig. 4a–c). The Sic1 levels were considerably lower compared to wild-type Sic1 during the arrest, as in the case of the T173S mutation. This suggests that the extended intracomplex degron pathway efficiently competes with the diversional path. However, because T173S-Sic1 does not undergo diversional termination, the profile apparently declines more, when compared with 3xN-Sic1, and, despite stronger intracomplex competition, still has some probability for termination due to intact T173.

In addition, besides the borderline α-factor arrest, we performed experiments in a slow-growth glycerol–ethanol media to test whether similar CDK activity leakage was universal for situations with prolonged G1. Indeed, we found that T173S-Sic1 displays a rather similar declining profile in cell cycles that underwent the change from rich to poor media as observed in α-factor pulse experiment (Supplementary Fig. 4d–f).

### Sic1 degradation mechanism at G1/S

When the Start decision is finally made, the balance between the degron and the diversional pathway is shifted toward the former and the accumulating S-CDK will meet more G1-CDK-primed N-terminal sites to finalize the degradation switch, while the MAPK Fus3 pathway targeting the diversional site T173 is switched off^47–49^. The model of Sic1 phosphorylation at G1/S, starting with G1-CDK priming, and followed by Cks1-dependent process where free S-CDK finalizes the degron phosphorylation^1,24^ is supported by the finding that the timing of Sic1 degradation was delayed in mutated versions affecting the G1-CDK dependent priming steps, Cks1 docking, and free S-CDK docking (the VLLPP, T2,5S-VLLPP, 9SP, and RXL mutants). We observed fast post-Start accumulation of Sic1 in case of the VLLPP, T2,5S-VLLPP, and 9SP mutations (Fig. 5i, j, Supplementary Fig. 3g, 4g, h). We can exclude general transcriptional activation as no *SIC1* transcriptional upregulation at Start has been observed previously^16,50,51^. We can speculate that the wave of Clb5 synthesis at Start is also accompanied with more Sic1 transported to the nucleus as part of the inhibitory complex^52^. However, in case of wild-type Sic1, the degradation starts immediately at Start and the post-Start accumulation is not observed.

**Fig. 6:**
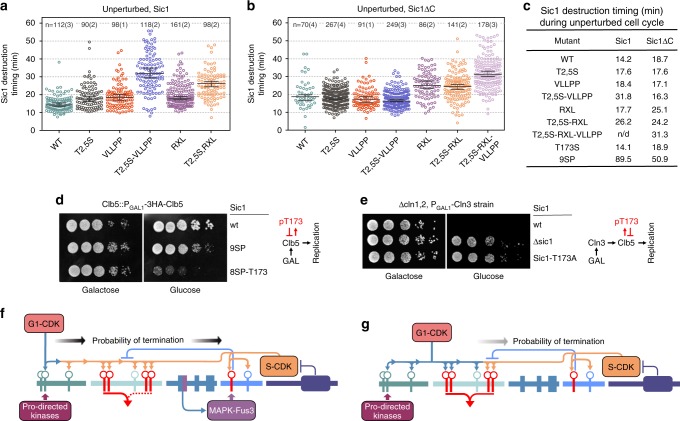
The intracomplex phosphorylation connects G1-CDK priming and S-CDK activity. **a**, **b** Plots showing the distributions of Sic1 destruction timing values of individual cells in case of unperturbed cell cycle for strains expressing mutated variants of either full-length Sic1-EGFP in **a** or Sic1ΔC-EGFP in **b**. In case of Sic1ΔC-EGFP strains, endogenous Sic1 was expressed in the background. The median value along with 95% confidence intervals are denoted by black lines on the plot. The number of individual cells (*X*) observed over a number of individual colonies (*Y*) is given in form *n* = *X*(*Y*). The values for mother and daughter cells separately and the additional information are presented in Supplementary Table 1 and in the Source data file for Fig. 6. **c** A table of measured Sic1 destruction timing median values for the indicated strains. **d** A viability assay of strains with sic1::sic1(9SP) and sic1::sic1(8SP-T173) genomic replacements and conditional GAL1-CLB5 overexpression. **e** A viability assay of strains with sic1-deletion and sic1::sic1(T173A) genomic replacement in cln1,2-deletion background and conditional GAL1-CLN3 overexpression. The experiments for panels ‘d’ and ‘e’ were performed twice with similar result. **f**, **g** Schemes showing the difference in diversional termination mechanism for the borderline pheromone arrest (**f**), and for G1/S transition (**g**). Source data are provided as a Source Data file.

To analyze whether the S-CDK catalyzed intracomplex process has a role in Sic1 degradation also in freely cycling cells, we analyzed the same set of mutants both in case of full-length Sic1 (Sic1) and C terminally truncated non-inhibitory Sic1 (Sic1ΔC) in an unperturbed cell cycle (without α-factor). In the case of full-length Sic1, the T2,5S, VLLPP, T2,5S-VLLPP, and RXL mutations all delayed the degradation timing (Fig. 6a, c). In contrast, these mutations, except the RXL mutation, showed no effect on timing in experiments with non-inhibitory Sic1ΔC (Fig. 6b, c, Supplementary Fig. 5a–c). In addition, the degradation of non-inhibitory Sic1ΔC is delayed compared to full-length Sic1 (Fig. 6c). Thus, the priming phosphorylation by G1-CDK likely becomes important through the intracomplex mechanism, as degradation of full-length Sic1 is delayed by T2,5S and VLLPP mutations, but this is not the case for Sic1ΔC. As the C-terminal inhibitory domain does not affect the phosphorylation rate of Sic1 by G1-CDK^26^, and the VLLPP mutation affects both full-length Sic1 and Sic1ΔC phosphorylation^1,46^, but only the full-length Sic1 showed the effect on VLLPP in vivo, one can conclude that S-CDK is the major driving force behind Sic1ΔC degradation. Moreover, the presence of the G1-CDK priming site T5 in Sic1ΔC was not important, possibly because the alternative priming site T33 is a good target for S-CDK RXL-specific phosphorylation^1^. On the other hand, the contribution of both VLLPP and RXL docking motifs and T5 priming site to degradation of full-length Sic1 indicates that G1- and S-CDK act cooperatively to promote Sic1 degradation. Taken together, we can conclude that the Sic1 degradation mechanism at G1/S may involve three major components: (i) priming by G1-CDK, (ii) an intracomplex step driven by inhibited S-CDK, and (iii) the positive feedback-driven extra-complex step driven by free emerging S-CDK.

In stress-free conditions, the T173S mutation did not affect Sic1 degradation timing but resulted in consistently lower Sic1 levels in case of full-length Sic1, indicating that the declining M-CDK and accumulating S-CDK signal leak was prevented via intracomplex phosphorylation of T173 by CDK and diversional termination (Supplementary Figs. 5c, d, 6a). This effect of Sic1 levels was not observed in case of Sic1ΔC suggesting that it is dependent on the inhibitor complex (Supplementary Fig. 6b).

In a previous study with 9AP-Sic1 in *Δclb5* strain, the shutdown of *GAL-CLB5* expression was found to be lethal^53^. Strikingly, 9SP-Sic1 in similar conditions is viable while 8SP–T173–Sic1 is not (Fig. 6d). This would suggest that T173 intracomplex phosphorylation by S-CDK becomes lethal, when the CDK threshold for Sic1 degradation is shifted up due to distributive (non-processive) Cks1-independent mode of multisite phosphorylation of 9SP-Sic1, as shown by us previously^2^. Because T173 did not affect the inhibition potency (Fig. 1d), this result additionally suggests that inhibited S-CDK could not degrade 8SP–T173–Sic1, as the balance of competing intracomplex routes is shifted in favor of diversional phosphorylation. Furthermore, another in vivo experiment was performed, to additionally show the importance of T173 for preventing the leak of Clb5, and consequently for holding the Sic1 barrier to keep the arrest. It is known that a strain where all the G1 cyclins are suppressed is inviable, and that deletion of SIC1 rescues the viability of these cells. Our experiment, however, shows that T173A-SIC1 also rescues these cells, which suggests that Clb5 leaks in the case of T173A (Fig. 6e).

**Fig. 7:**
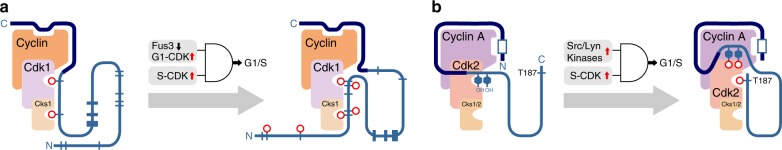
Regulation of mammalian p27 and yeast Sic1 is functionally analogous. **a**, **b** The intracomplex phosphorylation and subsequent release of CDK activity is regulated by AND gate-like logic. **a** In case of yeast Sic1, the increase of proliferation signal G1-CDK, and decrease of mating signal Fus3 prime Sic1 for inhibited S-CDK, which executes the Sic1 degradation and triggers the S-phase. **b** Analogously, in case of mammalian p27, the tyrosine kinases as proliferation signals prime the intracomplex phosphorylation for inhibited S-phase CDK complex.

The fact that Sic1 degradation timing was not affected by the T173S mutation in rich media suggests that the lock prevents leakage before the Start, to keep the Sic1 at necessary levels as a barrier for G1/S. However, when the Start decision has been made (50% Whi5 nuclear exit), preventing the leak is not a priority any more, and rapidly accumulating G1-CDK efficiently primes the N-terminal sites in Sic1, and the diversional lock is overrun because the probability of pT173-dependent termination is low due to the fewer number of steps required for S-CDK to reach the degrons (Fig. 6f, g). On the other hand, G1-CDK is not able to drive this switch alone, thereby creating an AND gate-like system that operates when sufficient levels of both G1- and S-CDK have been accumulating for G1/S transition (Fig. 7a, b). However, in a slow-growth medium (glycerol–ethanol) with a longer Start to G1/S time, the effect of T173S was also observed on Sic1 degradation timing, thus indicating that the S-CDK signal leak may have started to have an impact (Supplementary Fig. 4f). Furthermore, the T2,5S mutation in combination with the T173S balanced the two competing pathways (Supplementary Fig. 4e, f), additionally demonstrating the phenomenon of the balance between N-terminal and diversional phosphorylation routes.

## Discussion

In the present study, we made an attempt to explain a design paradox of the CKI-CDK double-negative feedback mechanism: high phosphorylation specificity of CDK toward CKI may compromise tight control over G1, and cause a phosphorylation-dependent degradation leak of CKI, thereby making it difficult to maintain the G1 state. We observed that high intracomplex phosphorylation activity manifested in processive phosphorylation of Sic1 within the inhibitory complex. As in human CKI p27, intracomplex phosphorylation by CDK mediated degradation of the inhibitor^54–57^.

We describe here mechanism of two competing intracomplex phosphorylation routes within the Sic1–Clb5–Cdk1–Cks1 inhibitory complex: the diversional site pT173 is able to prevent the leak via the alternative degron phosphorylation route. We also demonstrate that this diversional lock can be strengthened by extra input from MAPK Fus3 in case of pheromone pathway activity, a functionally overlapping role of Sic1 and Far1 to allow robust G1 arrest^58^. On the other hand, after the Start decision, the lock is overrun because the balance between the two routes shifts in favor of the N-terminal degron route due to efficient priming phosphorylation by rapidly accumulating G1-CDK.

This study reveals yet another complexity level of multisite phosphorylation mechanisms of CDK targets. Intricate network of phosphorylation sites and linear docking motifs along the disordered sections of Sic1 enable differential processing of at least four kinases with alternative input sites: G1-CDK as a primer for the N-terminal sites at G1/S transition, S-CDK as driver of priming-dependent phosphorylation of the degron route, and also as a diversionary input at T173, MAPKs as external inputs for the diversionary site T173, and a bulk of proline-directed kinases as constantly acting primers of site T5 (Fig. 6f).

In a functionally analogous system in mammalian cells, the intrinsic flexibility of inhibitor p27 in the complex with Cdk2/Cyclin A enables its phosphorylation by mitogenic tyrosine kinases, which lead to the opening of the active site on CDK that carries out intracomplex degron site (T187) phosphorylation^17,54,55,57,59–63^. In this case, however, the problem of leakiness is solved by orthogonality of tyrosine and serine–threonine kinase (CDK) prevents degron phosphorylation by the tyrosine kinase and enables a conditional AND gate-like trigger of G1/S only when both kinase signals are individually present above critical thresholds. Tyrosine kinase signaling is not as highly evolved in yeast as in higher eukaryotes, and the role of proliferation signals in yeast is fulfilled by the G1-CDK complexes, which link the cell cycle Start to the cell size control system, or alternatively, by the decline of MAPK Fus3 activity upon release from mating arrest (Fig. 7a, b). The individual inputs alone would not trigger the AND gate: G1-CDK is unable to meet the two-degron requirement^1^, and the intracomplex S-CDK route alone would be terminated at pT173. Thus, less orthogonal kinase inputs would require a more complex processing system, while the effort of developing highly orthogonal inputs pays off as they provide a chance to use a simpler system.

## Methods

### Yeast strain construction

All used yeast strains are described in Supplementary Table 2. Deletions, gene replacements, and tagging of endogenous loci was performed using PCR-based homologous recombination methods^64,65^. For quantification of endogenous Whi5, Sic1, and Clb5 proteins the corresponding loci were recombined at 3′ end by PCR-derived mCherry, EGFP or Citrine tag ORF and the selection marker cassette^64,65^. Similar approach was used to tag Sic1 with C-terminal 6xHA for western blotting analysis in Supplementary Fig. 2a^65^. To introduce genomic replacements of *SIC1* gene with mutated versions, PCR-derived DNA fragments were used to generate exact genomic replacements over *sic1::URA3* using 5FOA selection. To generate strains for Sic1ΔC time-lapse microscopy, pRS306 vector with *P*_*SIC1*_-Sic1ΔC-EGFP cassette was integrated into *URA3* locus of the strain with *WHI5-mCherry:SpHis5* background. To generate strains for Sic1ΔC western blotting, the pRS316 vector with *P*_*ADH1*_-Sic1ΔC-3xHA cassette was used. For Supplementary Fig. 2c, the genomic gene deletion strains were used^66^. For Supplementary Fig. 2a, the pRS306 plasmid carrying *P*_*SIC1*_-Sic1ΔC-6xHA was used for integration into *URA3* locus. Copy number of integrated pRS306 constructs carrying Sic1ΔC-EGFP was assessed by measuring quantitative levels of EGFP expression and qPCR analysis of genomic DNA. Please see Supplementary Table 3 for all plasmids and Supplementary Table 4 for the primers used in this study.

### Protein purification

Recombinant N-terminally 6xHis-tagged Sic1 proteins were expressed from pET28a(+) plasmid in *E. coli BL21RP* and purified using cobalt^2+^ affinity chromatography. Cyclins were copurified together as stoichiometric complexes with active Cdk1 from Δ*sic1* yeast strain using tandem affinity purification (TAP)^1,67–69^. Cks1 was expressed and purified from *E. coli BL21RP* as described previously^70^. Swe1 was expressed and purified from yeast cells using TAP-tag affinity purification^67^.

### In vitro phosphorylation and dephosphorylation assays

The general composition of phosphorylation reaction mixture was as follows: 50 mM HEPES-KOH pH 7.4, 5 mM MgCl_2_, 150 mM NaCl, 0.1% NP-40, 20 mM imidazole, 2% glycerol, 2 mM EGTA, 0.2 mg ml^−1^ BSA, 250 μM ATP, and 500 nM Cks1. Wild-type or phosphate-binding pocket mutant Cks1^71^ was added to the reactions containing Cks1, and mock buffer was used for reactions without Cks1. For quantitative intracomplex phosphorylation assays, 0.02 μM Clb5–Cdk1 complex and 1 μM recombinant Sic1 was used. The reactions containing a reference substrate were supplemented with 1 μM bovine histone H1 (Sigma-Aldrich). For phosphate labeling, 0.5 μCi μl^−1^ radiometric label [γ-^32^P]-ATP (Hartmann Analytic) was added 5 min after the enzyme was added to the reaction buffer containing unlabeled ATP. Finally, the reaction was initiated by adding Sic1 or Sic1 and histone H1 mixture.

For quantitative phosphorylation assays presented in Figs. 3a, e, 4b and Supplementary Fig. 3c, d, h, the enzyme concentration was 2 nM and substrate protein was 1 μM. For quantitative phosphatase assay in Fig. 3f, first, the intracomplex phosphorylation of Sic1 with [γ-^32^P]-ATP label was performed as described above. After 6 min of phosphorylation reaction, 5 mM DDT and 6 mM MnCl_2_ was supplemented followed by addition of recombinant lambda phosphatase (Santa Cruz Biotechnology) in 50 U µl^−1^ final concentration. All reactions were carried out at room temperature and aliquots were removed into SDS–PAGE sample buffer.

For separating the phospho-isoforms of Sic1, 10% SDS–PAGE supplemented with 100 μM Phos-tag and MnCl_2_ was used according to the instructions from the manufacturer^28^. Unless noted otherwise, 10% SDS–PAGE was used. The identification of different Sic1 phospho-isoforms using Phos-tag SDS–PAGE is described in ref. ^1^

After SDS–PAGE, gels were subjected to vacuum drying and subsequent autoradiography using Amersham BAS-IP MS 2040 E screen, Biomolecular Imager Typhoon 5 (Amersham), and ImageQuant TL software (Amersham, version 8.1).

To estimate Clb5–Cdk1–Cks1 intracomplex activity in units of phosphate numbers per Sic1 molecule bound to the complex, the same concentration of CDK complex was phosphorylated by Swe1 in a parallel reaction for reference. As Cdk1 is monophosphorylated by Swe1, the Cdk1 ^32^P signal in the reference mixture corresponds to one phosphate added to Sic1 per inhibited complex.

### Mass spectrometry

To determine the phosphorylation sites of Sic1 intracomplex, 10 µg of purified 6xHis-Sic1 recombinant protein was phosphorylated using 20 nM Clb5–Cdk1–Cks1. After incubating the kinase reaction for 6 min at room temperature the reaction was stopped in SDS–PAGE sample buffer. The proteins were separated using 10% SDS–PAGE, the gels were stained with Coomassie brilliant blue G-250 (Sigma), and Sic1 protein band was excised from the gels. 1 µg of Sic1 was used for the analysis. Subsequent in-gel digestion, peptide separation, MS analysis and Mascot search was performed as described previously^2^.

### Western blotting and immunoprecipitation

For western blotting, 10–30 µg of pelleted cells were snap frozen in liquid nitrogen and subsequently lysed by silica bead grinding in lysis buffer containing urea. The lysate was cleared by centrifugation, the supernatant was normalized over total protein content determined by Bradford assay (BioRad), subjected for gel electrophoresis. For detecting HA epitope the blotting was carried out by using anti-HA (mouse HA.11 clone 16B12 from Covance, cat# MMS-101R 1:1000 dilution) and anti-mouse-IgG HRP conjugated antibody (Labas AS, custom made, 1:10,000 dilution). For detecting Cdk1 anti-Cdk1 antibody (goat anti-Cdc28 antibody yC-20, from Santa Cruz Biotechnology, cat# sc-6709, 1:1000 dilution) and anti-goat-IgG HRP conjugated antibody (Labas AS, custom made, 1:10,000 dilution) were used. For electroblotting of Phos-tag SDS–PAGE gels, a dry system iBlot (Invitrogen) was used. Conjugated HRP signal was detected using Pierce™ ECL Western Blotting Substrate (Thermo Fisher Scientific).

To carry out the co-immunoprecipitation of Sic1–Clb5–TAP–Cdk1–Cks1 complex, recombinant *CLB5–TAP* in 2-μm plasmid was overexpressed using *GAL1* promoter. The expression was induced in strains containing endogenously expressed Sic1–6HA or T173A–Sic1–6HA. For co-immunoprecipitation, Clb5–TAP purification using 5–7 g of cell pellet was performed as described above. The presence of Sic1–6HA and Cks1 in final eluate was assessed by western blotting using anti-HA (mouse HA.11 clone 16B12 from Covance, 1:1000 dilution) or anti-Cks1 (rabbit antibody from Labas AS, custom made, 1:1000 dilution), and anti-mouse-IgG or anti-rabbit-IgG HRP conjugated antibodies (Labas AS, custom made, 1:10,000 dilution).

For quantitative western blotting, the enhanced chemiluminescence (ECL) signal was detected by ImageQuant RT ECL Imager 3088E camera (Amersham) with highest resolution and in variable shutter speed to secure all pixels were below signal saturation threshold. The uncropped images of Typhoon scans, ImageQuant RT ECL images, and all quantitated areas on these images are presented in Source Data files together with the signal values obtained for each area.

### Culture conditions

For western blotting, log-phase cultures grown in selective synthetic complete (SSC) or synthetic complete (SC) media were used. For α-factor synchronization, >95% pure synthetic α-factor peptide (Stork Biochemicals) as a 10 mg ml^−1^ (~5.9 mM) stock solution in DMSO was used. The final α-factor concentration was 0.59 µM for *bar1*^*−*^ strains and 5.9 µM for *BAR1*^*+*^ strains. For arresting cells with α-factor, the appropriate peptide concentration was added at OD 0.3 for 3 h. G1 arrest was confirmed by visually observing *shmoo* morphology. To induce G1 arrest in *Δcln1,2 cln3::P*_*GAL1,10*_*-CLN3* background strains via repression of *CLN3* mRNA synthesis, 2% glucose was added for 3.5 h.

For time-lapse microscopy, log-phase yeast culture grown in SC media was used. Before subjecting cells to Y04C type CellASIC ONIX microfluidic plates (EMD Millipore) the culture was sonicated until cell aggregates were separated into single cells. Cells were loaded into the growth chip as described in CellASIC online protocol using SC media supplemented with 2% glucose. The media containing 240 or 2 nM α-factor used in the pulse experiments, or media containing 2% glycerol and 1% ethanol instead of glucose, for long G1 experiments, was supplied from separate wells. For the α-factor pulse and glycerol–ethanol experiments, a previously described protocol was used^42,44^. Before the switch to α-factor or glycerol–ethanol media, cells were allowed to grow unperturbed under the flow of SC 2% glucose media for at least 60 min. Constant media flow at pressure 5 psi was used through the experiment.

In the yeast viability assays in Fig. 6d, e, cultures were grown in YPG media to log-phase. The cultures were serially diluted to attain cell densities 10^6^, 5 × 10^5^, 10^5^, 10^4^, 5 × 10^3^ cells ml^−1^. Subsequently, 5 µl of culture was used for spotting.

### Quantitative time-lapse microscopy

Time-lapse imaging of cells growing in the microfluidic chips was carried out with Zeiss Axio Observer Z1 inverted microscope using motorized stage and phase contrast imaging combined with Colibri LED module to excitate mCherry, EGFP or Citrine fluorescence. For excitation of EGFP, 470 nm LED for 15 ms, for Citrine 505 nm LED for 700 ms and for mCherry 555 nm LED for 750 ms were used at 25% power. For experiments containing Sic1-EGFP, a maximum of 10 fields of view were collected. For imaging, Hamamatsu Orca ER CCD Monochrome Microscope Camera was used. The microscopy setup was similar to Dončić et al.^43^. The images were collected and exported in raster image formats (.JPG) by using ZEN software (Zeiss, version 2.3, blue edition) and were subjected to automated cell tracking and fluorescence signal quantitation process using custom MATLAB (compatible with version 2016a) scripts as described previously^72^. For Sic1-EGFP signal quantification, minor custom changes were made.

The fluorescence time-lapse profiles were subjected to manual assessment for cell tracking and imaging quality and further timing measurement^43,45^. For quantification, the maximal fluorescence plateau level was measured using the aid of a digital ruler. Next, the half-maximal value of the signal and corresponding point in the time axis was determined. Finally, the program delivers the corresponding time value automatically into an output table. All the software used for microscopy image quantification and analysis are available upon request.

### Statistics and reproducibility

At least two independent experiments were conducted for all kinase assays and western blots. The number of quantitative replicates used, as well as the raw data for quantification of all conducted experiments are available in Source Data files. For all kinase assays and quantitative western blots, the mean values were derived from at least two replica experiments.

In live-cell microscopy, data of each strain were collected from at least two independent cell colonies. On the Sic1 degradation timing distribution plots, the median values as well as 95% confidence interval values are shown. Also, the 95% confidence intervals of the medians are summarily presented in Supplementary Table 1.

### Reporting summary

Further information on research design is available in the Nature Research Reporting Summary linked to this article.

## Supplementary information

Supplementary Information

Reporting Summary

## Data Availability

All data or biological materials will be made available upon request from corresponding author Mart Loog (mart.loog@ut.ee). The source data underlying Figs. 1–6 are provided as a Source Data file.

## Source Data

Source data

## Code availability

All custom made scripts for single-cell image segmentation, tracking, and quantification will be made available upon request from corresponding author Mart Loog (mart.loog@ut.ee).

## References

[1] Koivomagi M (2011). Cascades of multisite phosphorylation control Sic1 destruction at the onset of S phase. Nature.

[2] Koivomagi M (2013). Multisite phosphorylation networks as signal processors for Cdk1. Nat. Struct. Mol. Biol..

[3] Valk E (2014). Multistep phosphorylation systems: tunable components of biological signaling circuits. Mol. Biol. Cell.

[4] Van Roey K, Gibson TJ, Davey NE (2012). Motif switches: decision-making in cell regulation. Curr. Opin. Struct. Biol..

[5] Stern B, Nurse P (1996). A quantitative model for the cdc2 control of S phase and mitosis in fission yeast. Trends Genet..

[6] Gutierrez-Escribano P, Nurse P (2015). A single cyclin-CDK complex is sufficient for both mitotic and meiotic progression in fission yeast. Nat. Commun..

[7] Swaffer MP, Jones AW, Flynn HR, Snijders AP, Nurse P (2016). CDK substrate phosphorylation and ordering the cell cycle. Cell.

[8] Swaffer MP, Jones AW, Flynn HR, Snijders AP, Nurse P (2018). Quantitative phosphoproteomics reveals the signaling dynamics of cell-cycle kinases in the fission yeast *Schizosaccharomyces pombe*. Cell Rep..

[9] Kamenz J, Ferrell JEJ (2017). The temporal ordering of cell-cycle phosphorylation. Mol. Cell.

[10] Enserink JM, Kolodner RD (2010). An overview of Cdk1-controlled targets and processes. Cell Div..

[11] Örd M, Loog M (2019). How the cell cycle clock ticks. Mol. Biol. Cell.

[12] Örd M (2019). Multisite phosphorylation code of CDK. Nat. Struct. Mol. Biol..

[13] Örd M, Venta R, Möll K, Valk E, Loog M (2019). Cyclin-specific docking mechanisms reveal the complexity of M-CDK function in the cell cycle. Mol. Cell.

[14] Holt LJ (2009). Global analysis of Cdk1 substrate phosphorylation sites provides insights into evolution. Science.

[15] Mendenhall MD (1993). An inhibitor of p34CDC28 protein kinase activity from *Saccharomyces cerevisiae*. Science.

[16] Schwob E, Bohm T, Mendenhall MD, Nasmyth K (1994). The B-type cyclin kinase inhibitor p40SIC1 controls the G1 to S transition in S. cerevisiae. Cell.

[17] Huang Y, Yoon M-K, Otieno S, Lelli M, Kriwacki RW (2015). The activity and stability of the intrinsically disordered Cip/Kip protein family are regulated by non-receptor tyrosine kinases. J. Mol. Biol..

[18] Lengronne A, Schwob E (2002). The Yeast CDK inhibitor Sic1 prevents genomic instability by promoting replication origin licensing in late G1. Mol. Cell..

[19] Verma R, Feldman RM, Deshaies RJ (1997). SIC1 is ubiquitinated in vitro by a pathway that requires CDC4, CDC34, and cyclin/CDK activities. Mol. Biol. Cell.

[20] Feldman RMR, Correll CC, Kaplan KB, Deshaies RJ (1997). A complex of Cdc4p, Skp1p, and Cdc53p/cullin catalyzes ubiquitination of the phosphorylated CDK inhibitor Sic1p* physiological ubiquitination reactions require E3s, and whether all E3s will participate directly in both substrate. Cell.

[21] Verma R (1997). Phosphorylation of Sic1p by G1 Cdk required for its degradation and entry into S phase. Science.

[22] Jakobson L (2016). Natural variation in arabidopsis Cvi-0 accession reveals an important role of MPK12 in guard cell CO_2_ signaling. PLoS Biol..

[23] Verma R, McDonald H, Yates JR3rd, Deshaies RJ (2001). Selective degradation of ubiquitinated Sic1 by purified 26S proteasome yields active S phase cyclin-Cdk. Mol. Cell.

[24] Yang X, Lau K-Y, Sevim V, Tang C (2013). Design principles of the yeast G1/S switch. PLoS Biol..

[25] Cantley, L., Hunter, T., Thorner, J., Sever, R. *Signal Transduction: Principles, Pathways, and Processes* (CSHL Press, New York, 2016).

[26] Venta R, Valk E, Koivomagi M, Loog M (2012). Double-negative feedback between S-phase cyclin-CDK and CKI generates abruptness in the G1/S switch. Front. Physiol..

[27] Schneider BL, Yang QH, Futcher AB (1996). Linkage of replication to start by the Cdk inhibitor Sic1. Science.

[28] Kinoshita E, Yamada A, Takeda H, Kinoshita-Kikuta E, Koike T (2005). Novel immobilized zinc(II) affinity chromatography for phosphopeptides and phosphorylated proteins. J. Sep. Sci..

[29] Escote X, Zapater M, Clotet J, Posas F (2004). Hog1 mediates cell-cycle arrest in G1 phase by the dual targeting of Sic1. Nat. Cell Biol..

[30] Zinzalla V, Graziola M, Mastriani A, Vanoni M, Alberghina L (2007). Rapamycin-mediated G1 arrest involves regulation of the Cdk inhibitor Sic1 in *Saccharomyces cerevisiae*. Mol. Microbiol..

[31] Moreno-Torres M, Jaquenoud M, Peli-Gulli M-P, Nicastro R, De Virgilio C (2017). TORC1 coordinates the conversion of Sic1 from a target to an inhibitor of cyclin-CDK-Cks1. Cell Discov..

[32] Moreno-Torres M, Jaquenoud M, De Virgilio C (2015). TORC1 controls G1-S cell cycle transition in yeast via Mpk1 and the greatwall kinase pathway. Nat. Commun..

[33] McGrath DA (2013). Cks confers specificity to phosphorylation-dependent CDK signaling pathways. Nat. Struct. Mol. Biol..

[34] Hodge A, Mendenhall M (1999). The cyclin-dependent kinase inhibitory domain of the yeast Sic1 protein is contained within the C-terminal 70 amino acids. Mol. Gen. Genet..

[35] Cross FR, Yuste-Rojas M, Gray S, Jacobson MD (1999). Specialization and targeting of B-type cyclins. Mol. Cell.

[36] Cross FR, Jacobson MD (2000). Conservation and function of a potential substrate-binding domain in the yeast Clb5 B-type cyclin. Mol. Cell. Biol..

[37] Takeda DY, Wohlschlegel JA, Dutta A (2001). A bipartite substrate recognition motif for cyclin-dependent kinases. J. Biol. Chem..

[38] Wohlschlegel JA, Dwyer BT, Takeda DY, Dutta A (2001). Mutational analysis of the Cy motif from p21 reveals sequence degeneracy and specificity for different cyclin-dependent kinases. Mol. Cell. Biol..

[39] Tyers M, Tokiwa G, Futcher B (1993). Comparison of the *Saccharomyces cerevisiae* G1 cyclins: Cln3 may be an upstream activator of Cln1, Cln2 and other cyclins. EMBO J..

[40] Mcinerny, C. J., Partridge, J. F., Mikesell, G. E., Creemer, D. P. & Breeden, L. L. A novel Mcml-dependent element CDC4S7 promoters activates M/Gi-specific transcription. *Genes Dev.* **11**, 1277–1288 (1997). 10.1101/gad.11.10.1277 9171372

[41] Elion EA, Brill JA, Fink GR (1991). FUS3 represses CLN1 and CLN2 and in concert with KSS1 promotes signal transduction. Proc. Natl Acad. Sci. USA.

[42] Skotheim JM, Di Talia S, Siggia ED, Cross FR (2008). Positive feedback of G1 cyclins ensures coherent cell cycle entry. Nature.

[43] Doncic A, Falleur-Fettig M, Skotheim JM (2011). Distinct interactions select and maintain a specific cell fate. Mol. Cell.

[44] Doncic A, Skotheim JM (2013). Feedforward regulation ensures stability and rapid reversibility of a cellular state. Mol. Cell.

[45] Doncic A (2015). Compartmentalization of a bistable switch enables memory to cross a feedback-driven transition. Cell.

[46] Koivomagi M (2011). Dynamics of Cdk1 substrate specificity during the cell cycle. Mol. Cell.

[47] Oehlen LJ, Cross FR (1994). G1 cyclins CLN1 and CLN2 repress the mating factor response pathway at Start in the yeast cell cycle. Genes Dev..

[48] Strickfaden SC (2007). A mechanism for cell-cycle regulation of MAP kinase signaling in a yeast differentiation pathway. Cell.

[49] Repetto MV (2018). CDK and MAPK synergistically regulate signaling dynamics via a shared multi-site phosphorylation region on the scaffold protein Ste5. Mol. Cell.

[50] Knapp D, Bhoite L, Stillman DJ, Nasmyth K (1996). The transcription factor Swi5 regulates expression of the cyclin kinase inhibitor p40SIC1. Mol. Cell. Biol..

[51] Amoussouvi A (2018). Transcriptional timing and noise of yeast cell cycle regulators-a single cell and single molecule approach. NPJ Syst. Biol. Appl..

[52] Rossi RL, Zinzalla V, Mastriani A, Vanoni M, Alberghina L (2005). Subcellular localization of the cyclin dependent kinase inhibitor Sic1 is modulated by the carbon source in budding yeast. Cell Cycle.

[53] Cross FR, Schroeder L, Bean JM (2007). Phosphorylation of the Sic1 inhibitor of B-type cyclins in *Saccharomyces cerevisiae* is not essential but contributes to cell cycle robustness. Genetics.

[54] Galea CA (2008). Role of intrinsic flexibility in signal transduction mediated by the cell cycle regulator, p27 Kip1. J. Mol. Biol..

[55] Chu I (2007). p27 phosphorylation by Src regulates inhibition of cyclin E-Cdk2. Cell.

[56] Ou L (2011). Incomplete folding upon binding mediates Cdk4/cyclin D complex activation by tyrosine phosphorylation of inhibitor p27 protein. J. Biol. Chem..

[57] Ou L, Waddell MB, Kriwacki RW (2012). Mechanism of cell cycle entry mediated by the intrinsically disordered protein p27(Kip1). ACS Chem. Biol..

[58] Pope PA, Pryciak PM (2013). Functional overlap among distinct G1/S inhibitory pathways allows robust G1 arrest by yeast mating pheromones. Mol. Biol. Cell.

[59] Tsytlonok, M. et al. Dynamic anticipation by Cdk2/Cyclin A-bound p27 mediates signal integration in cell cycle regulation. *Nat. Commun.* **10**, 1676 (2019). 10.1038/s41467-019-09446-w PMC6459857 30976006

[60] Das RK, Huang Y, Phillips AH, Kriwacki RW, Pappu RV (2016). Cryptic sequence features within the disordered protein p27Kip1 regulate cell cycle signaling. Proc. Natl Acad. Sci. USA.

[61] Grimmler M (2007). Cdk-inhibitory activity and stability of p27Kip1 are directly regulated by oncogenic tyrosine kinases. Cell.

[62] Barberis M (2005). The yeast cyclin-dependent kinase inhibitor Sic1 and mammalian p27Kip1 are functional homologues with a structurally conserved inhibitory domain. Biochem. J..

[63] Barberis M (2012). Molecular systems biology of Sic1 in yeast cell cycle regulation through multiscale modeling. Adv. Exp. Med. Biol..

[64] Longtine MS (1998). Additional modules for versatile and economical PCR-based gene deletion and modification in *Saccharomyces cerevisiae*. Yeast.

[65] Janke C (2004). A versatile toolbox for PCR-based tagging of yeast genes: new fluorescent proteins, more markers and promoter substitution cassettes. Yeast.

[66] Giaever G (2002). Functional profiling of the *Saccharomyces cerevisiae* genome. Nature.

[67] Puig O (2001). The tandem affinity purification (TAP) method: a general procedure of protein complex purification. Methods.

[68] Ubersax JA (2003). Targets of the cyclin-dependent kinase Cdk1. Nature.

[69] McCusker D (2007). Cdk1 coordinates cell-surface growth with the cell cycle. Nat. Cell Biol..

[70] Reynard GJ, Reynolds W, Verma R, Deshaies RJ (2000). Cks1 is required for G(1) cyclin-cyclin-dependent kinase activity in budding yeast. Mol. Cell. Biol..

[71] Bourne Y (2000). Crystal structure and mutational analysis of the *Saccharomyces cerevisiae* cell cycle regulatory protein Cks1: implications for domain swapping, anion binding and protein interactions. Structure.

[72] Doncic A, Eser U, Atay O, Skotheim JM (2013). An algorithm to automate yeast segmentation and tracking. PLoS ONE.

