# Supplementary material for: A processive phosphorylation circuit with multiple kinase inputs and mutually diversional routes controls G1/S decision
Source: Nat Commun. 2020 Apr 15;11:1836. doi: 10.1038/s41467-020-15685-z (PMC7160111; doi:10.1038/s41467-020-15685-z)
Supplement: Supplementary file 1 — Supplementary Information [file 41467_2020_15685_MOESM1_ESM.pdf]

Supplementary Information

A processive phosphorylation circuit with multiple kinase inputs and mutually diversional routes controls G1/S decision

Supplementary Note 1.

Multiple sequence alignment of assortment of budding yeasts orthologous Sic1 protein regions containing T173 phosphorylation site.

The alignment contains *S. cerevisiae* Sic1 protein sequence at positions 165-185. Homologous T173 position is marked by red dot above. Positions with highlighted background represent over 85% amino acid similarity. Below the alignment is the conservation score for each position, ranging from 0 to 10, where 0 indicates no conservation and 10 (denoted by asterisk) indicates 100% identity. The multiple sequence alignment is constructed by using MUSCLE program[1] and manual editing.

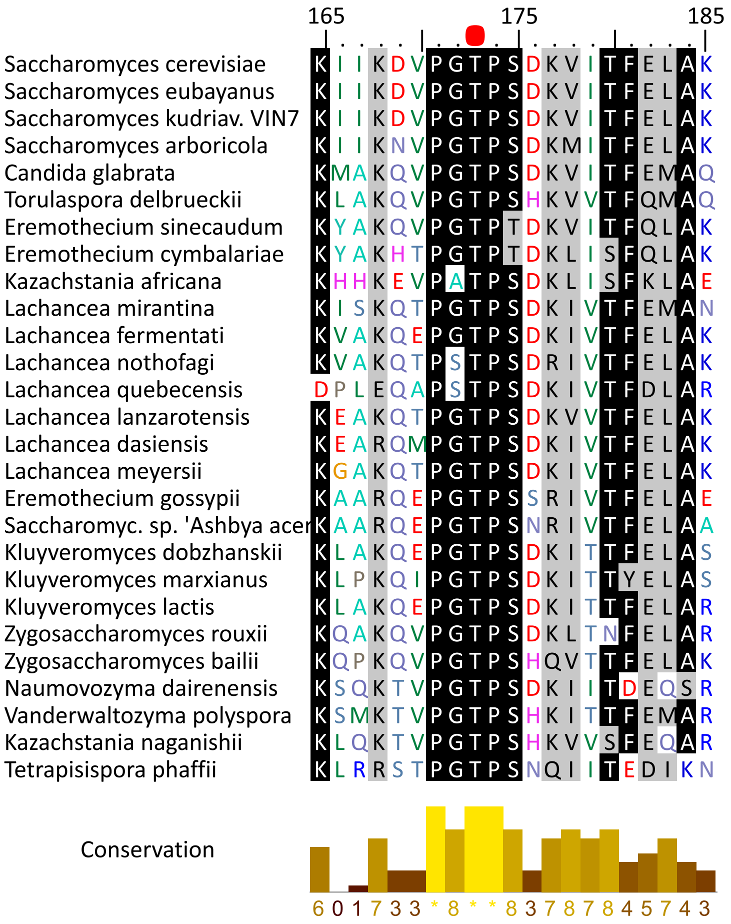

Supplementary Note 2

Docking connections in Sic1 phosphorylation

To understand the importance of intracomplex phosphorylation, Fus3 and G1-CDK in regulation of Sic1 during an elongated G1, different docking connections were interrupted or created by specific mutations in Sic1 (**Fig. 5l**). As pT173-Cks1 interaction terminates the intracomplex phosphorylation (**connection 1 in Fig. 5l**), T173S mutation leads to increased phosphorylation of the N-terminal sites within the inhibitory complex and decreased Sic1 levels during the elongated G1 (**Fig. 5h**). The degron sites are phosphorylated in Cks1-dependent manner using the N-terminal TP-sites for docking (**connection 2 in Fig. 5l**) (Kõivomägi 2013[2]). Mutation of the TP sites to SP in 9SP-Sic1 leads to decreased Cks1-mediated phosphorylation of the degron sites and stabilization of Sic1 (**Fig. 5i**). 50% on T5 is phosphorylated in G1 (**Supplementary Fig. 2d**) and the pT5 can prime phosphorylation of other N-terminal sites via Cks1 docking (**connection 3 in Fig. 5l**). The T2S T5S mutation abolishes this interaction and leads to higher Sic1 levels during the G1 arrest (**Supplementary Fig. 3g**). G1-CDK phosphorylates N-terminal sites in Sic1 using the VLLPP docking motif (Kõivomägi 2011[3], Bhaduri and Pryciak 2011[4]) (**connection 4 in Fig. 5l**), priming the N terminus for subsequent phosphorylation by S-CDK. Mutation of the VLLPP docking sites leads to slightly increased Sic1 leves in G1 and delayed degradation after Start (**Supplementary Fig. 3g**). Combined mutation of T2,5S and VLLPP interrupts the effect of priming phosphorylation by G1-CDK and the T5 kinase (**connection 5 in Fig. 5l**) and results in increased Sic1 stability in G1 and a large delay in Sic1 degradation after Start (**Fig. 5j**). MAPK Fus3 docking site overlaps with ^{89}RXL in Sic1 and the docking promotes T173 phosphorylation by Fus3 (**Supplementary Fig. 3h**, **connection 6 in Fig. 5l**). Mutation of the RXL motifs results in decreased Sic1 levels in pheromone-induced elongated G1 (**Fig. 5k**), presumably due to decreased Fus3 input to T173 phosphorylation. As the G1-CDK input, detected by the mutation of VLLPP docking motif, was found to decrease Sic1 stability in G1, we wanted to see if G1-CDK could be directed to prime the phosphorylation of the diversionary site T173 kinase (**connection 7 in Fig. 5l**). For this, we added G1-CDK specificity determinants proline in -2 and lysine in +2 position from the phosphorylation site to the site T173 (T173-2P+3KA: PGT^{173}PKA) (Kõivomägi 2011[3]). This mutation led to increased Sic1 levels in the α-factor pulse experiment (**Fig. 5k**), presumably due to increased T173 phosphorylation by G1-CDK.

Supplementary Note 3

Related to the halo assay of pheromone sensitivity in Suppl. Fig. 3a.

Comparison with the study Schwob E. & Nasmyth K. Genes Dev. 1993 Fig. 7[5]. In mentioned previous work the strains carrying WT-Sic1 in pGAL1-Clb5 background strain could not be arrested by alpha-factor. In present study Sup. Fig. 3a the strain carrying WT-Sic1 allele is arrested. The different result originates from the fact, that the strain used for the halo assay in previous study contained two copies of CLB5 under the GAL promoter and one copy of CLB5 under its endogenous promoter. In present study, however, we use a strain with only one copy of pGAL-CLB5, which means that the discrepancy can be explained by the fact that in the mentioned paper the pGAL1-Clb5 strain likely cannot be arrested by alpha-factor because of a 2-3-fold higher dose of Clb5 compared to our experiment.

Supplementary Figure 1

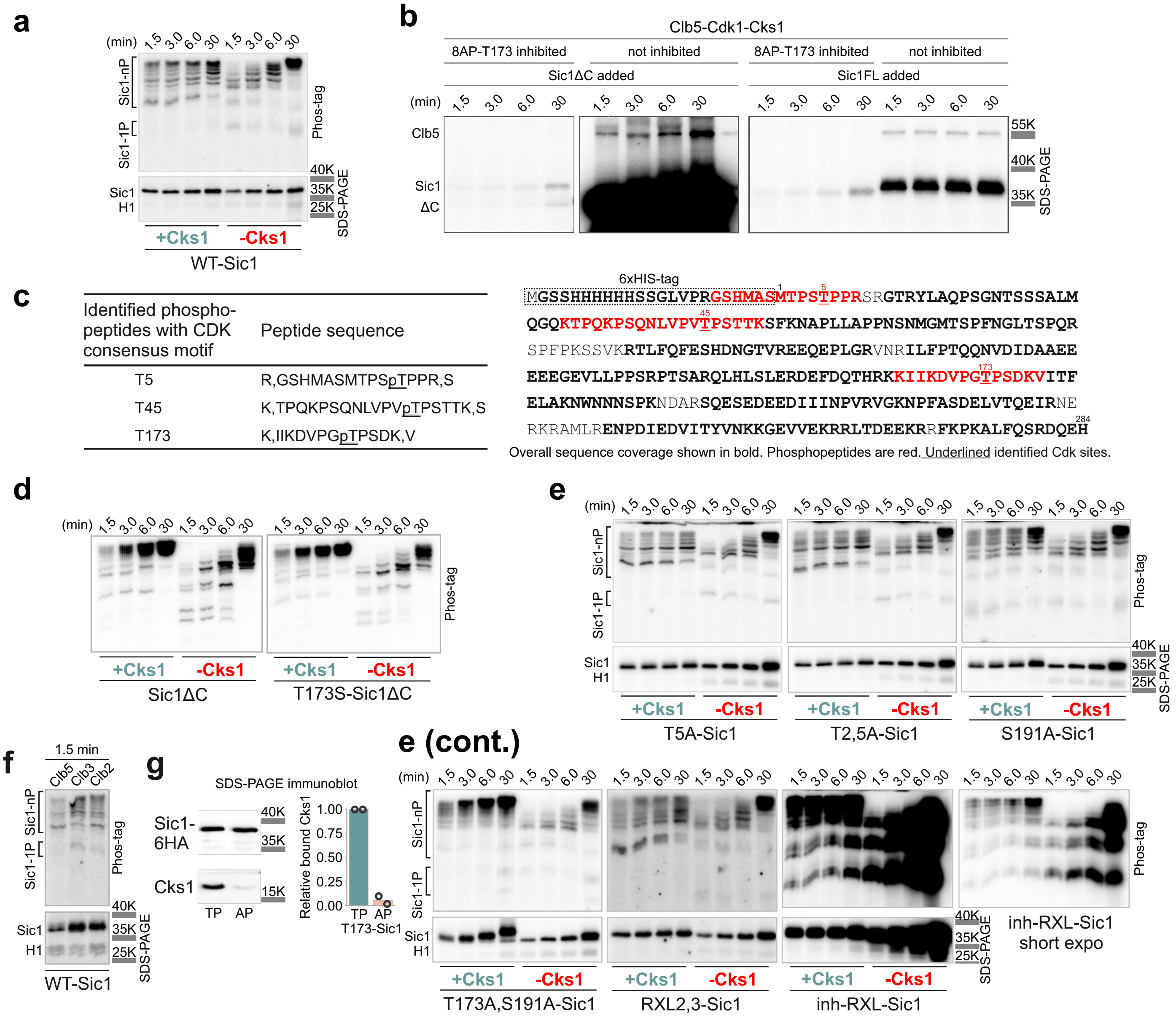

Supplementary Figure 1. Processive phosphorylation of Sic1 within the S-CDK-Sic1 inhibitory complex. a The intracomplex phosphorylation of Sic1 by Clb5-Cdk1 was followed in the presence or absence of Cks1. The phosphorylated forms were separated by Phos-tag SDS-PAGE followed by 32P autoradiography (top gel). Conventional SDS-PAGE was used in lower panels to separate an assay mixture where Histone H1 was included as an external substrate control. The experiment was repeated four times with similar results. **b** After formation of inhibitory complex containing Clb5-Cdk1-Cks1 and excess amount of 8AP-T173-Sic1, a Sic1 version where all CDK sites, except T173 were mutated to alanines, wild type full-length Sic1 or Sic1ΔC was added. The residual activity toward added Sic1 versions was over 30 000 times lower than that of uninhibited free enzyme towards Sic1ΔC. **c** A mass spectrometry analysis of Sic1 phosphorylation sites added by the intracomplex phosphorylation within the Clb5-Cdk1-Cks1-Sic1 complex. Three phosphopeptides containing Cdk1 consensus motifs (TP/SP) were detected, as highlighted on Sic1 sequence. **d** Multisite phosphorylation of non-inhibitory C-terminally truncated versions of Sic1 (Sic1ΔC and T173S-Sic1ΔC) using Clb5-Cdk1-Cks1 complex. **e** The intracomplex phosphorylation of different Sic1 phosphorylation site mutants was followed in the presence and absence of the phospho-adaptor Cks1 as in 'a'. The RXL2,3-Sic1 denotes the Sic1 version with mutations in both Clb5-specific docking sites in the docking module of Sic1 (Fig. 1c). The inh-RXL-Sic1 denotes a mutation in the C-terminal inhibitory domain known to reduce the inhibitory potency of Sic1[34]. **f** The intracomplex phosphorylation of WT-Sic1 by Clb5-,

Supplementary Figure 1 (continuing)

Clb3- and Clb2-Cdk1-Cks1 complexes in a similar assay as in panels 'a' and 'e'. **g** Western blotting analysis of the co-immunoprecipitation of Clb5-TAP-Cdk1-Cks1 from yeast extracts obtained from strains expressing wild type Sic1-6HA or T173A-Sic1-6HA. Left upper panel: the co-precipitated Sic1-6HA. Left bottom panel: the co-precipitated Cks1. Right panel: relative quantification of co-precipitated Cks1. Mean values presented in the chart by bars are obtained from two independent experiments, for which the results are plotted individually by the open circles. The experiments for panels 'b' and d-g were performed twice with similar result.

Supplementary Figure 2

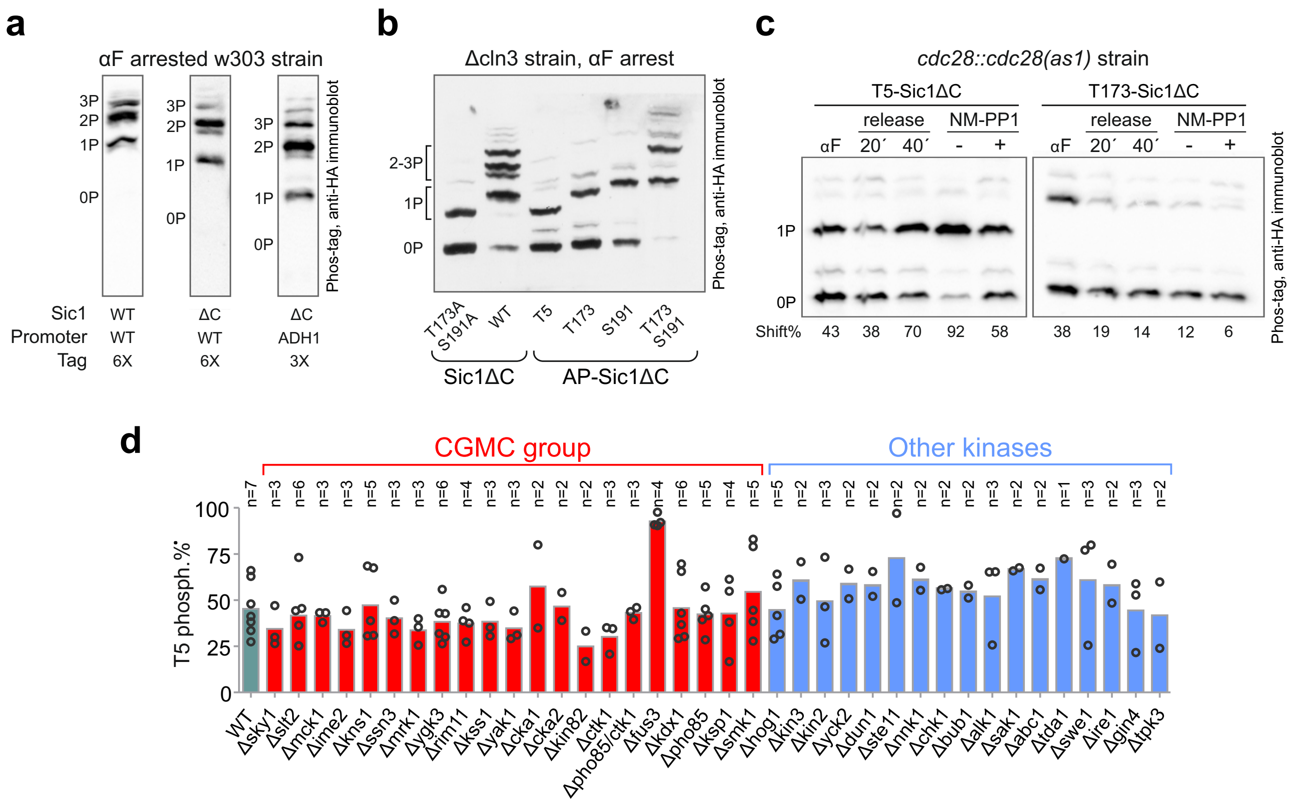

Supplementary Figure 2. Analysis of the phosphorylation pattern of Sic1 in G1 arrested cells. a Analysis of the phosphorylation patterns of Sic1 in pheromone arrested G1 cells with different HA-tagged versions of full length Sic1 and non-inhibitory Sic1ΔC under endogenous or ADH1 promoters using Phos-tag SDS-PAGE western blotting. Because neither the expression level of Sic1 nor the absence of the inhibitory domain affected the patterns, the other kinase activities rather than possible trace amounts of inhibited S-Cdk1 were responsible for the phosphorylations. **b** A Phos-tag Western blotting analysis of the phosphorylation of Sic1ΔC mutated versions in Δcln3 strain. In the four lanes, denoted with AP-Sic1ΔC, only the indicated phosphorylation sites were left unmutated, while the other CDK sites were mutated to alanines. **c** Phosphorylation of T5-Sic1ΔC and T173-Sic1ΔC after the release of cells from α-factor-induced G1 arrest in analog-sensitive Cdk1 strain 68 (Cdk1(as1)). After 40 minutes of the release 10 µM NM-PP1 Cdk1(as1) inhibitor was added, and aliquots were taken at indicated time points. This approach was used to eliminate the contribution of Cdk1 activity on T5 phosphorylation. After release of the cells expressing the T5-Sic1ΔC construct from G1 arrest, the shift of pT5 initially increased, due to the accumulating S-CDK activity, while after addition of the inhibitor, the shift dropped to the level characteristic for the G1 arrest. This suggests that the kinase activity responsible for roughly 50% steady-state phosphorylation level of T5 is constant during cell cycle, while the T173 shift was present only in pheromone arrested G1 state. The experiments for panels a-c were performed twice with similar result. **d** To search for kinases responsible for T5 phosphorylation, a set of kinase deletion strains[6] were analyzed for potential changes in the pT5-Sic1ΔC phosphorylation shift in α-factor arrested cells. Western blotting was performed on Phos-tag separated cell lysates and the values were calculated as percentage of quantified phosphorylated bands relatively to the sum of non-phosphorylated and phosphorylated bands. Mean values presented in the chart as bars are obtained from at least two independent experiments, for which the results are plotted individually by the open circles.

Supplementary Figure 3

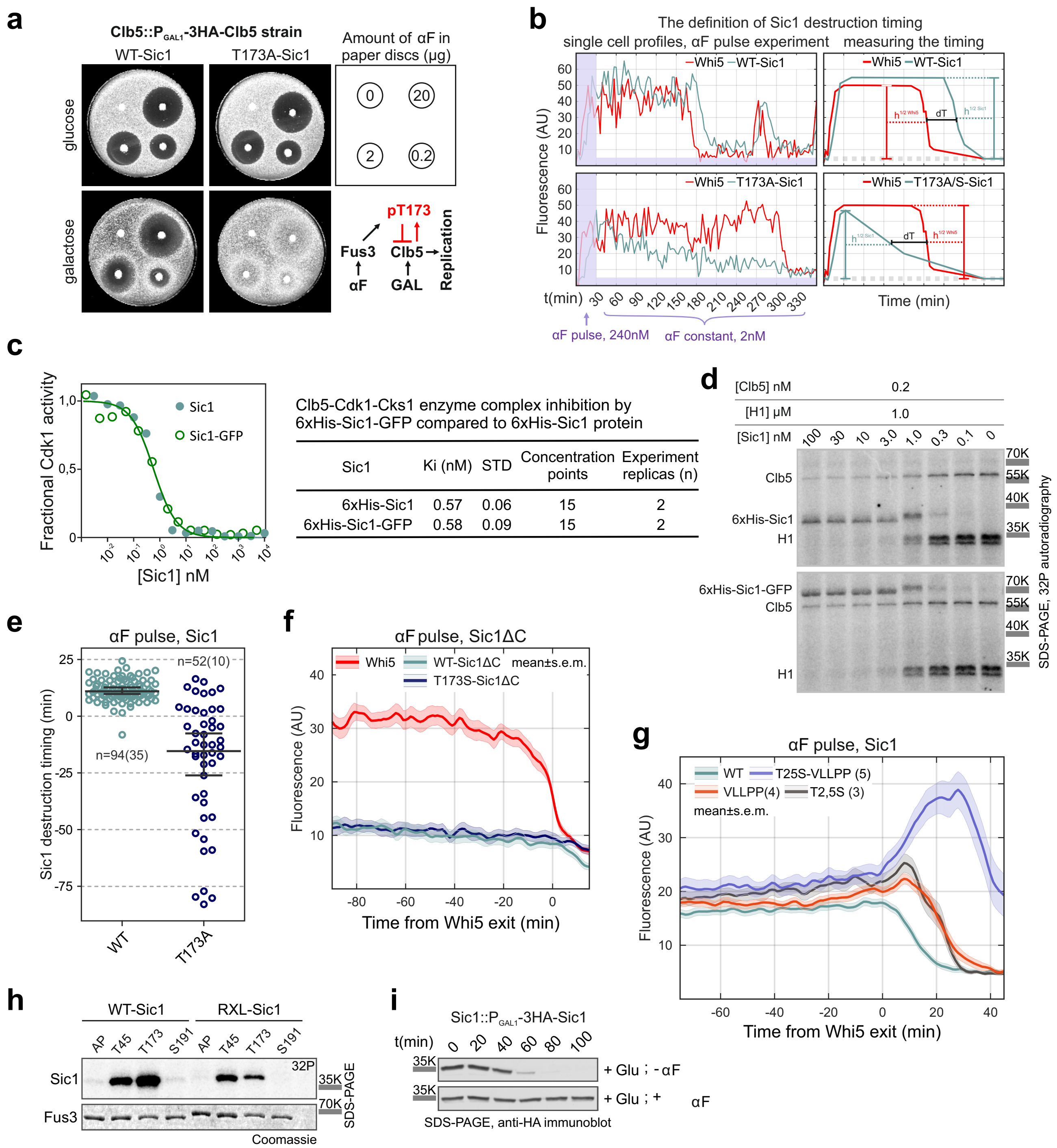

Supplementary Figure 3. In vivo analysis of the diversionary mechanism. a A halo assay of pheromone sensitivity in cells expressing Sic1 or T173A-Sic1 from endogenous locus and S-phase cyclin Clb5 under the GAL1 promoter. By immersing paper discs into α–factor solution and placing the discs on agar plates a radially diffusing gradient of the pheromone is created. Cell growth or inviability around the discs indicates the insensitivity or sensitivity to the pheromone pathway. The signaling scheme describes the role of pT173-Sic1 in pheromone arrest. For additional explanations please see Supplementary Note 3. **b** The left panels present examples of quantified fluorescence signals in strains expressing wild type Sic1-GFP and T173A-Sic1-GFP. The Sic1 destruction timing from Whi5 nuclear exit is measured as indicated schematically on the right panel. **c** Comparison of functionality of 6His-Sic1-GFP and 6His-Sic1 in a Clb5-Cdk1-Cks1 inhibition assay using Histone H1 as substrate. The plots obtained from two independent experiments were overlapping. The table presents Ki values calculated as IC50 of inhibition, based on the

Supplementary Figure 3 (continuing)

knowledge that the concentration of H1 used was considerably lower than measured KM for H1. **d** Comparison of functionality of 6His-Sic1-GFP and 6His-Sic1 in the standard intracomplex phosphorylation assay. Autoradiography of the SDS-PAGE is shown. **e** Plots showing the distributions of Sic1 destruction timing values of the individual cells from the Sic1-GFP and T173A-Sic1-GFP strains in the α-factor pulse experiment exemplified in panel 'b'. The median value along with 95% confidence intervals are denoted by black lines on the plot. The number of individual cells (X) observed over a number of induvidual colonies (Y) is given in form n=X(Y). **f** Mean fluorescence levels for wild type Sic1ΔC-GFP and T173S-Sic1ΔC-GFP in the α-factor pulse experiment. Wild type full-length Sic1 was expressed from its endogenous promoter in the background. The Whi5-mCherry is shown for the strain with the wild type Sic1ΔC-GFP only. The shaded areas represent ± s.e.m. **g** The mean fluorescence levels for T2,5S-Sic1-GFP, VLLPP-Sic1-GFP, T2,5S-VLLPP-Sic1-GFP, and WT-Sic1-GFP calculated from the α-factor pulse experiment. The shaded areas represent ± s.e.m. **h** Purified Sic1 constructs containing only single Fus3 sites, the T173-Sic1 and T45-Sic1, with the rest of S/TP sites mutated, were phosphorylated in initial velocity conditions using purified Fus3. Both autoradiography and the coomassie scans are shown. **i** A western blotting analysis of GAL-overexpressed Sic1-3HA in α-factor arrested cells. Glucose was added to shut down the transcription either in the presence or the absence of 600 nM α-factor. The experiments for panels 'a', 'c-d' and 'h-i' were performed twice with similar result.

Supplementary Figure 4

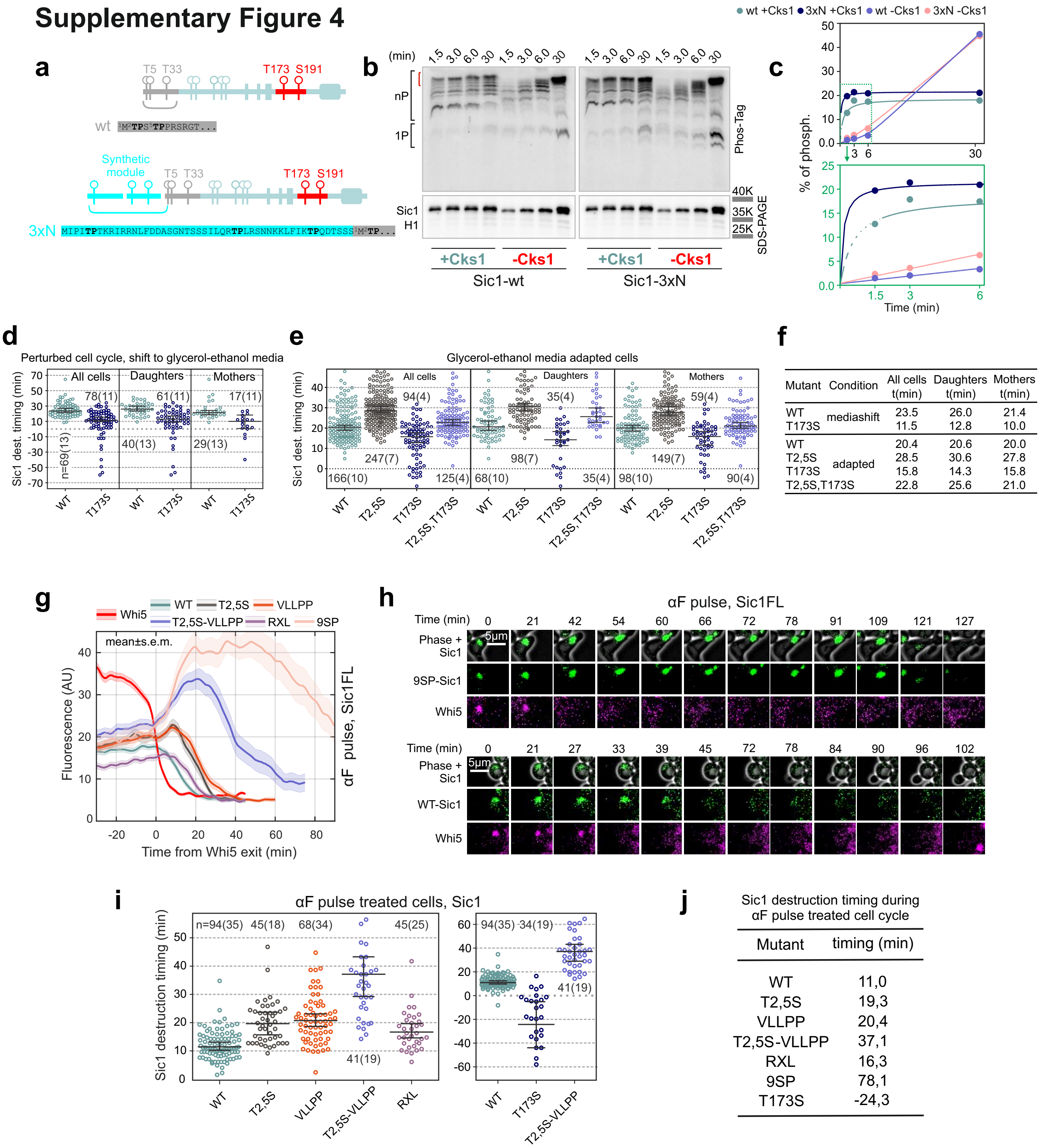

f

Mutant	Condition	All cells t(min)	Daughters t(min)	Mothers t(min)
WT	mediashift	23.5	26.0	21.4
T173S		11.5	12.8	10.0
WT	adapted	20.4	20.6	20.0
T2,5S		28.5	30.6	27.8
T173S		15.8	14.3	15.8
T2,5S,T173S		22.8	25.6	21.0

j Sic1 destruction timing during αF pulse treated cell cycle

Mutant	timing (min)
WT	11,0
T2,5S	19,3
VLLPP	20,4
T2,5S-VLLPP	37,1
RXL	16,3
9SP	78,1
T173S	-24,3

Supplementary Figure 4. G1-CDK and S-CDK dependent interactions control the Sic1 degradation. a Scheme of the Sic1 construct with added N-terminal phosphorylation modules (3xN-Sic1). **b** The purified 3xN-Sic1 and wild type Sic1 were used for intracomplex phosphorylation assay with Clb5-Cdk1, autoradiographs of Phos-tag SDS-PAGE are shown. **c** Quantified 32P signals of hyperphosphorylated forms from 'b'. The plots were obtained from two independent experiments were nearly overlapping. **d,e** Cells grown in microfluidic plate were shifted from 2% glucose media to poor media containing 2% glycerol + 1% ethanol. Plots showing the distributions of Sic1 destruction timing values of individual cells counted from timepoints starting from 50% of Whi5 nuclear exit for cells right after media shift (d) and for cells adapted (e) to media. The median values along with 95% confidence intervals are denoted by black lines on the plot. The number of individual cells (X) observed over a number of induvidual colonies (Y) is given in form n=X(Y). The values of confidence intervals are presented in Supplementary Table 1. **f** The summarizing table of Sic1

Supplementary Figure 4 (continuing)

destruction timing values from panels 'd' and 'e'. **g** Dynamics of the mean fluorescence levels for the indicated strains shown in the time window of G1/S transition (data from the panels 'd-f' of Figure 5). The Whi5 profile is shown for the strain with wild type Sic1-GFP. **h** Examples of microscopy time-lapse images for wild type Sic1-GFP and 9SP-Sic1-GFP. The scale bar indicates 5µm. The sample cells were chosen from the total set of 94 or 17 cells observed in alpha factor pulse experiments with WT-Sic1 and 9SP-Sic1 strains respectively. **i** Plots showing the distributions of Sic1 destruction timing values for individual cells. The median values along with 95% confidence intervals are denoted by black lines on the plot. The number of individual cells (X) observed over a number of induvidual colonies (Y) is given in form n=X(Y). **j** A table of measured Sic1 destruction timing values for the indicated strains in the α-factor pulse ex

Supplementary Figure 5

a

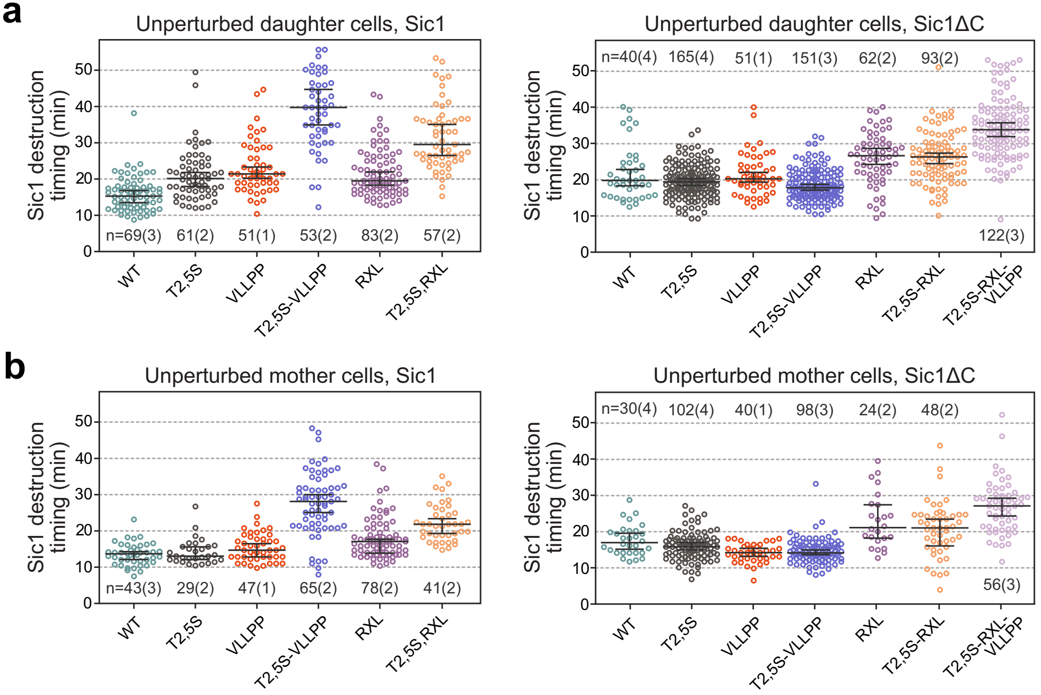

c

Sic1 destruction timing (min) during unperturbed cell cycle

	Daughter cells		Mother cells	
Mutant	Sic1	Sic1ΔC	Sic1	Sic1ΔC
WT	15.3	19.9	13.7	17.1
T2,5S	20.2	19.5	13.1	15.9
VLLPP	21.4	20.4	14.7	14.3
T2,5S-VLLPP	39.8	17.9	28.1	14.3
RXL	19.5	26.8	17.1	21.2
T2,5S-RXL	29.5	26.4	21.8	21.2
T2,5S-RXL-VLLPP	n/d	34.0	n/d	27.3
T173S	14.1	20.5	14.1	17.7
9SP	94.3	52.1	79.3	48.4

d

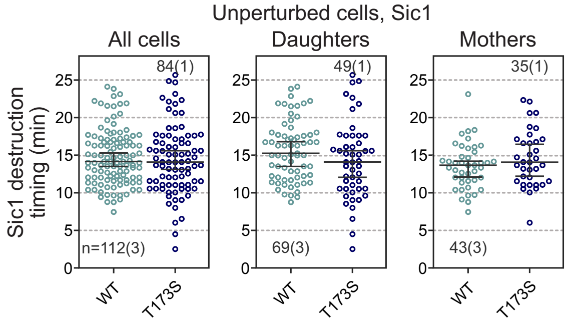

Supplementary Figure 5. The intracomplex phosphorylation affects both a mother and daughter cells. a,b Plots showing the distributions of Sic1 destruction timing values for individual cells of strains expressing different variants of either Sic1-GFP or Sic1ΔC-GFP in case of unperturbed cell cycle. Daughter (a) and mother (b) cells are indicated by separate plots. The median value along with 95% confidence intervals are denoted by black lines on the plot. The number of individual cells (X) observed over a number of induvidual colonies (Y) is given in form n=X(Y). **c** A table of measured Sic1 destruction timing median values for the indicated strains. For extended statistical information please see Supplementary Table 1. **d** Sic1 destruction timing values for wild type or T173S full-length Sic1-GFP in individual cells. The median value along with 95% confidence intervals are denoted by black lines on the plot. The number of individual cells (X) observed over a number of induvidual colonies (Y) is given in form n=X(Y).

Supplementary Figure 6

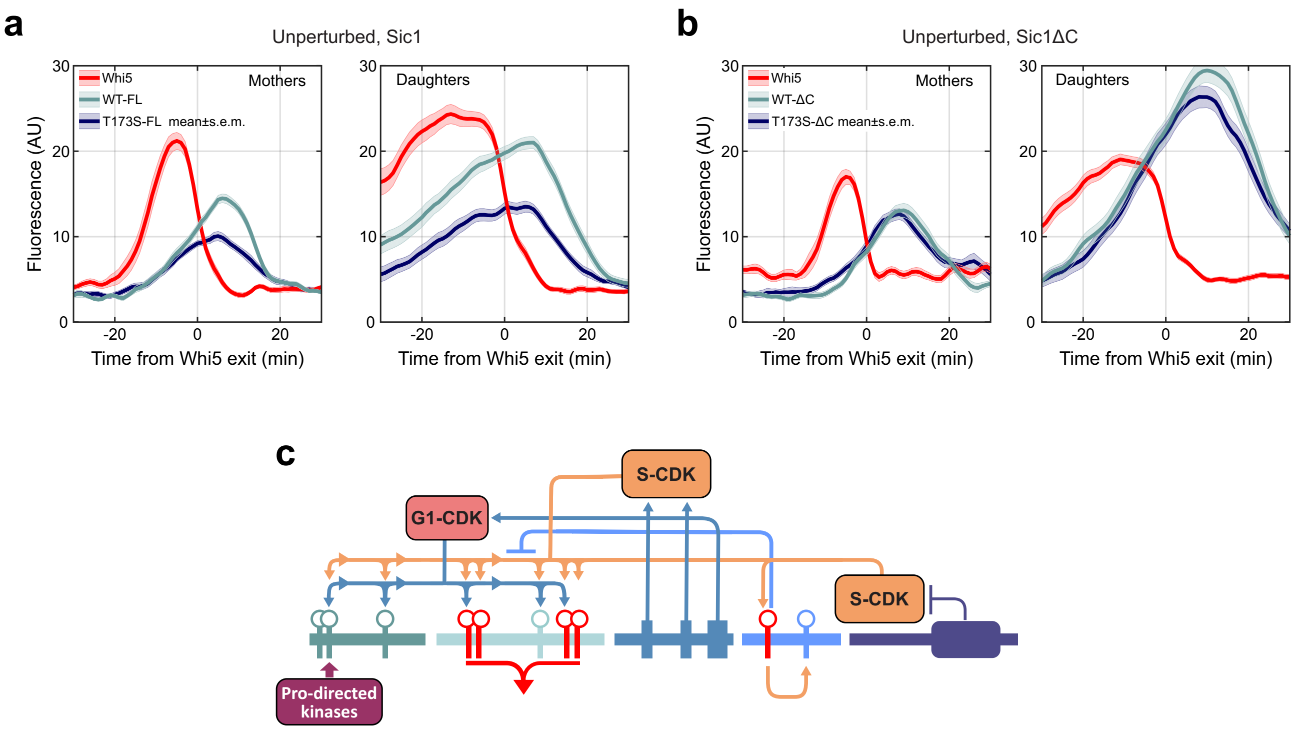

Supplementary Figure 6. The intracomplex phosphorylation of T173 by S-CDK is stabilizing Sic1 in unperturbed cell cycle. a,b Plots showing the mean nuclear fluorescence intensities of wild type or T173S-Sic1 in mother or daughter cells. The GFP-tagged Sic1 was either full-length (a) or non-inhibitory ΔC (b). Mean fluorescence levels for the time courses were calculated from values of individual cells undergoing unperturbed cell cycle. Whi5-mCherry levels are shown for both strains. The shaded areas represent ± s.e.m. **c** Scheme showing the crucial interactions involving the G1-CDK and S-CDK complexes that determine the Sic1 degradation dynamics at G1/S transition.

Supplementary Figure 7 Uncropped Original Scans

Figure 1d

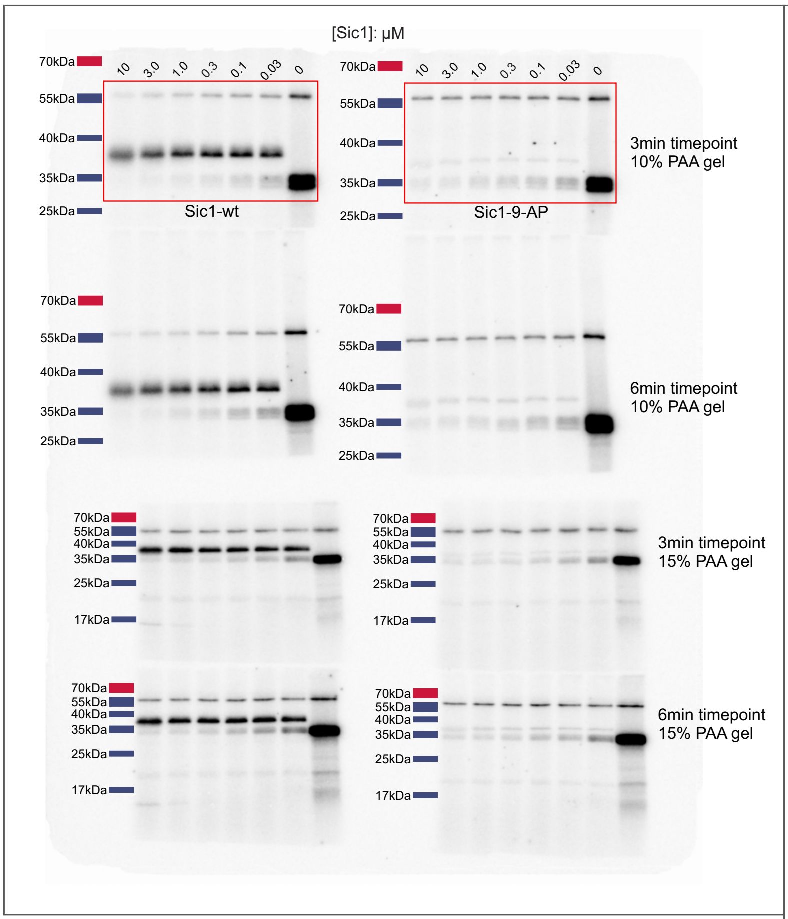

Figure 1e

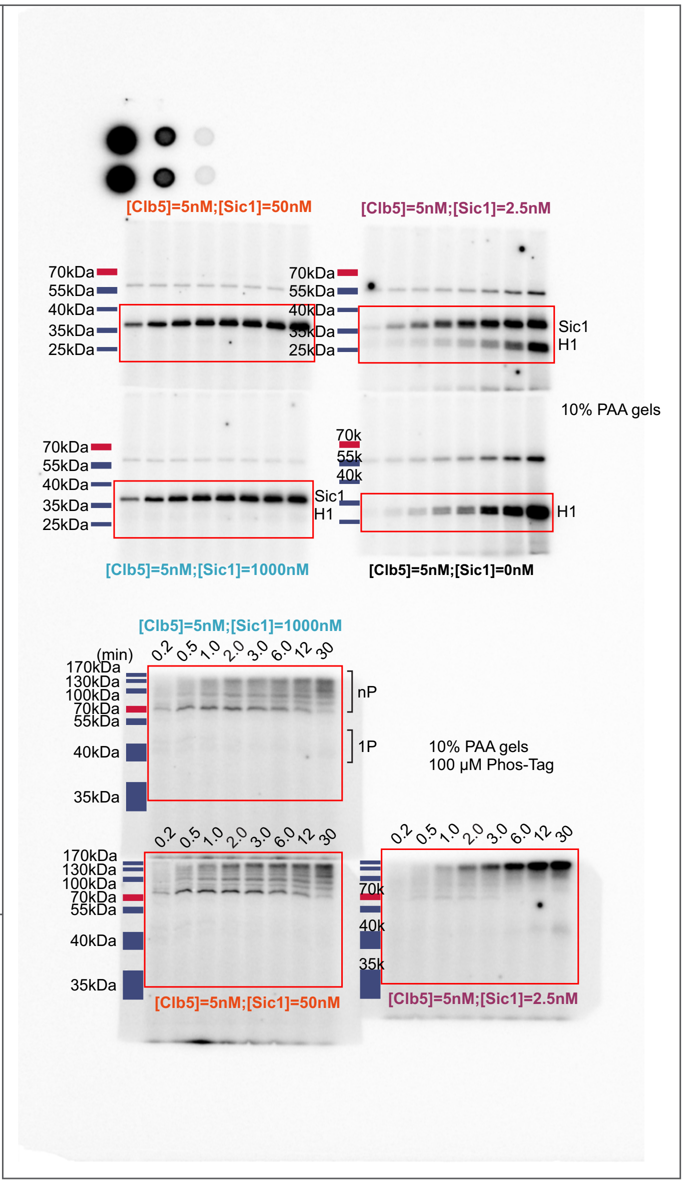

Supplementary Figure 7. Full scan images for Figure 1. Uncropped image of 32P-signal autoradiography by Typhoon Imager from the kinase assay electrophoresed in SDS-PAGE and Phos-Tag gels. The cropped areas for the figures are marked by the red line. Protein ladder used: PageRuler™ Prestained Protein Ladder, 10 to 180 kDa (Thermo Scientific, cat # 26617).

Figure 2a, Phos-Tag gels

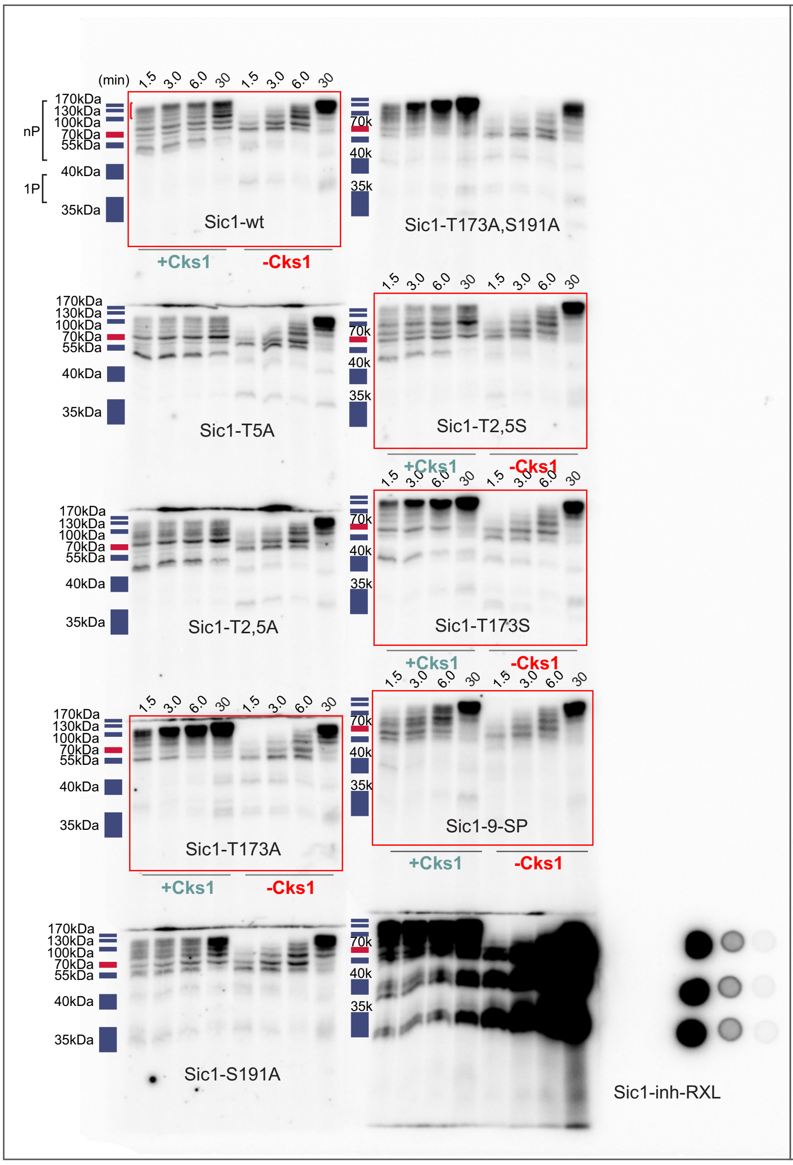

Figure 2a, SDS-PAGE gels

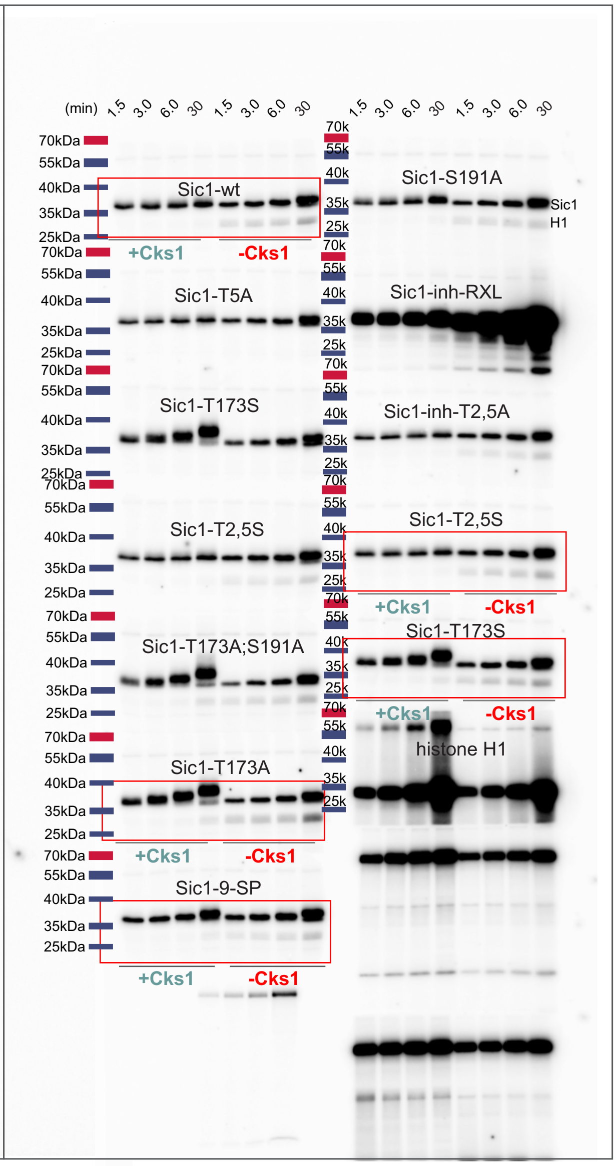

Figure 2c, Phos-Tag gels

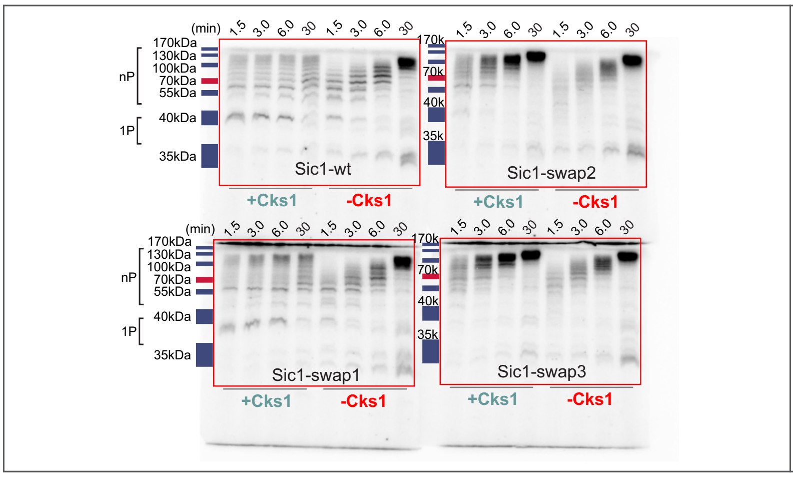

Figure 2c, SDS-PAGE gels

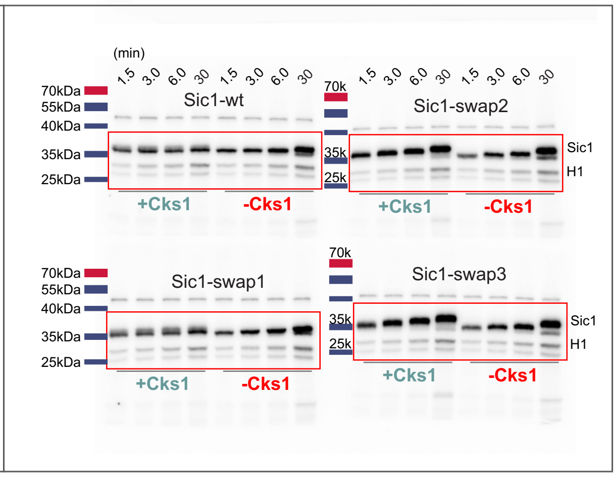

Supplementary Figure 7. Full scan images for Figure 2. Uncropped image of 32P-signal autoradiography by Typhoon Imager from the kinase assay electrophoresed in Phos-Tag (left) and SDS-PAGE (right) gels. The cropped areas for the figures are marked by the red line. Protein ladder used: PageRuler™ Prestained Protein Ladder, 10 to 180 kDa (Thermo Scientific, cat # 26617).

Figure 3a, SDS-PAGE gels

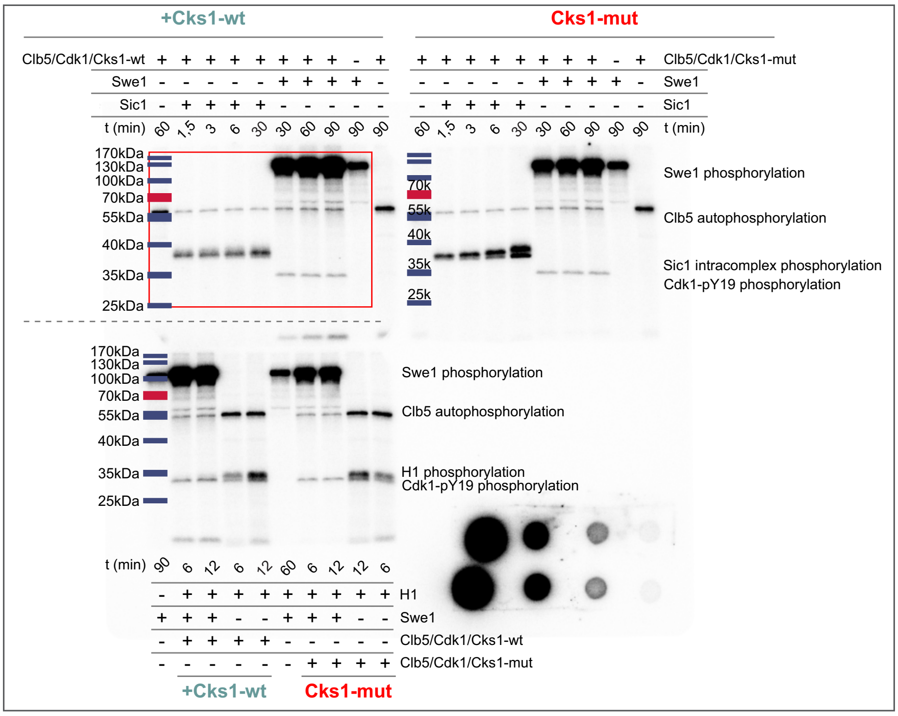

Figure 3e, SDS-PAGE and Phos-Tag gels

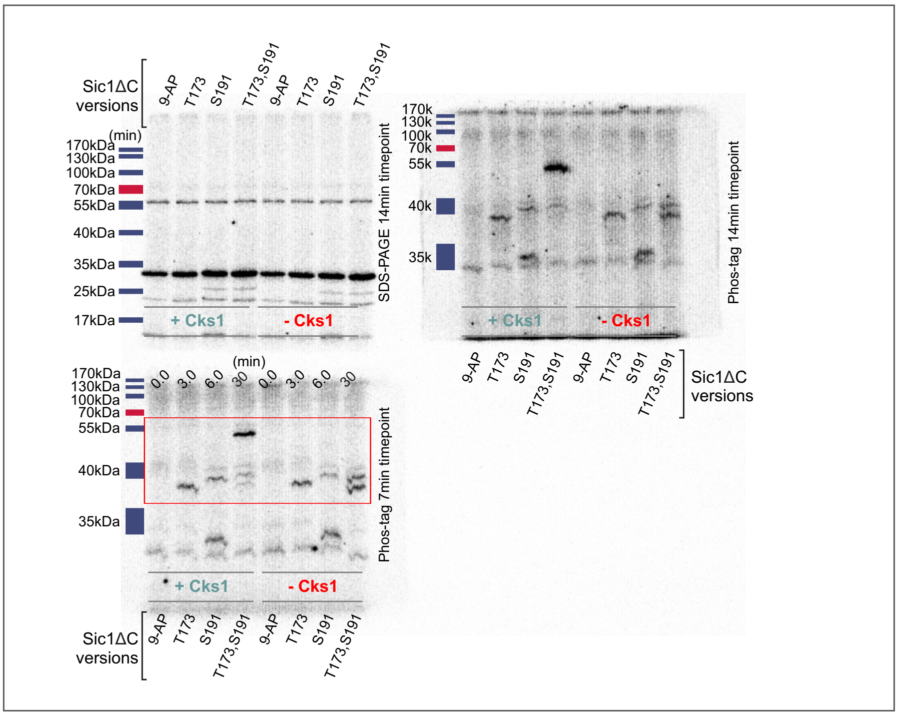

Supplementary Figure 7. Full scan images for Figure 3. Uncropped image of 32P-signal autoradiography by Typhoon Imager from the kinase assay electrophoresed in SDS-PAGE and Phos-Tag gels. In Fig. 3a all gels are SDS-PAGE gels. In Fig. 4e upper left gel is SDS-PAGE gel, upper right and bottom are Phos-Tag gels. The cropped areas for the figures are marked by the red line. Protein ladder used: PageRuler™ Prestained Protein Ladder, 10 to 180 kDa (Thermo Scientific, cat # 26617).

Figure 3b and 3d, SDS-PAGE gels

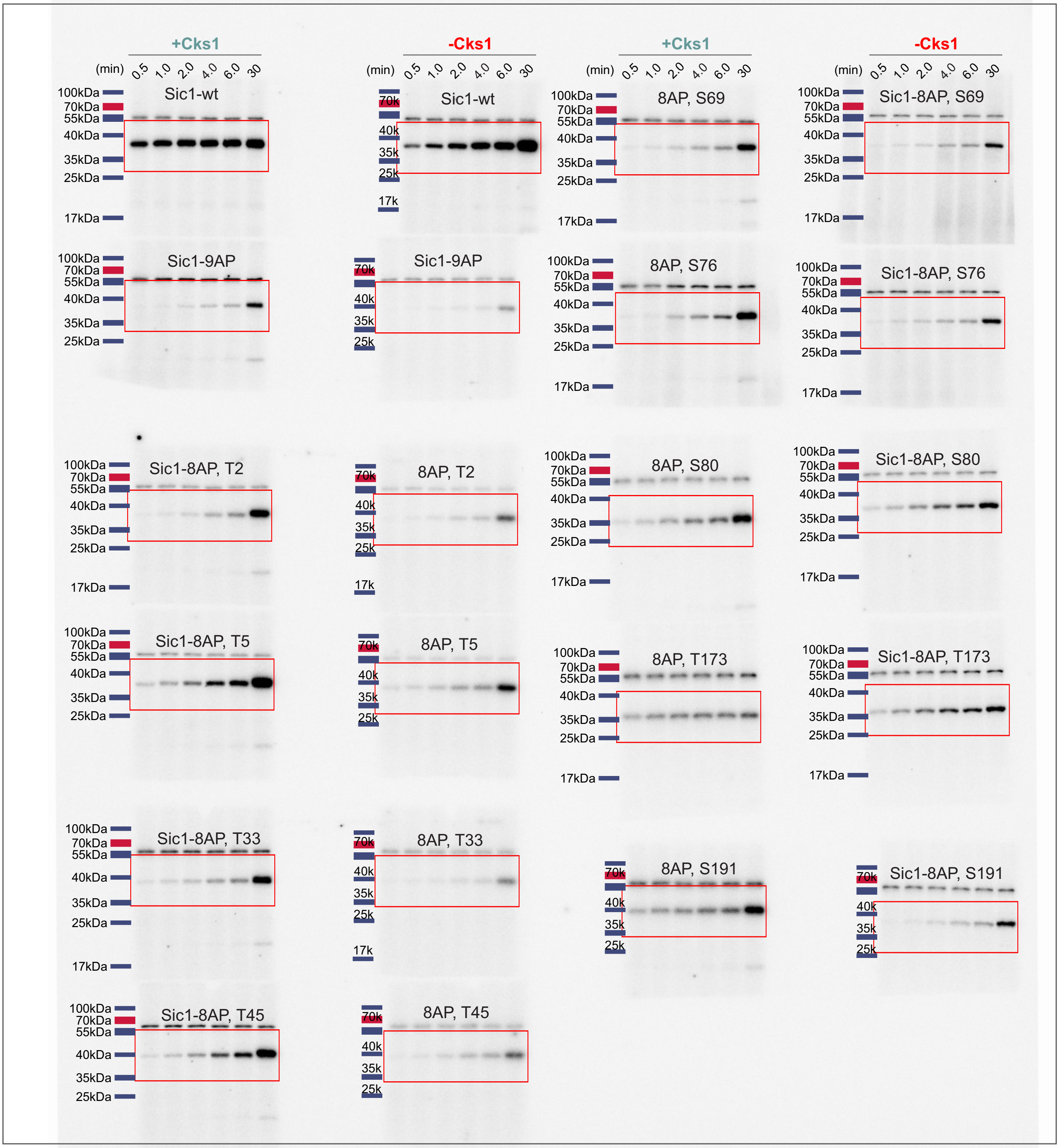

Supplementary Figure 7. Full scan images for Figure 3b and 3d. Uncropped image of 32P-signal autoradiography by Typhoon Imager from the kinase assay electrophoresed in SDS-PAGE gels. The cropped areas for the figures are marked by the red line. Protein ladder used: PageRuler™ Prestained Protein Ladder, 10 to 180 kDa (Thermo Scientific, cat # 26617).

Figure 4a. Immunoblot luminescence

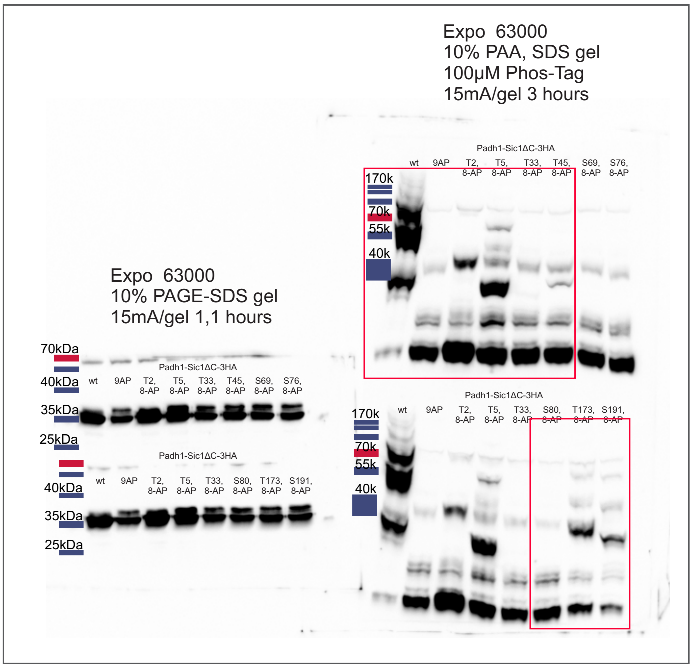

Figure 4b. 32P-signal autoradiography

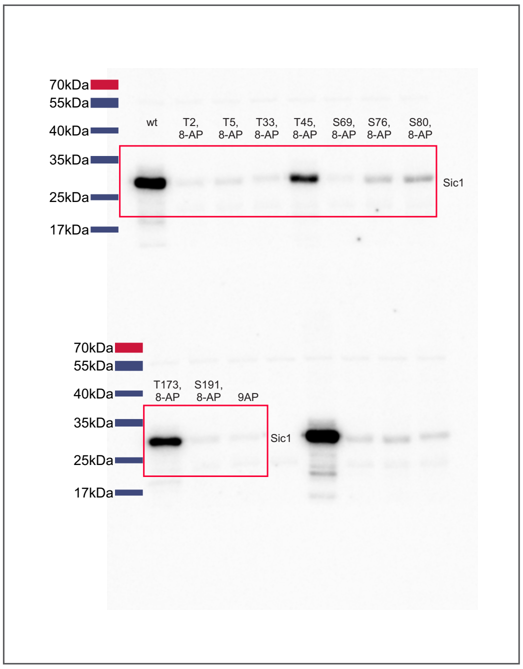

Figure 4c. Immunoblot luminescence

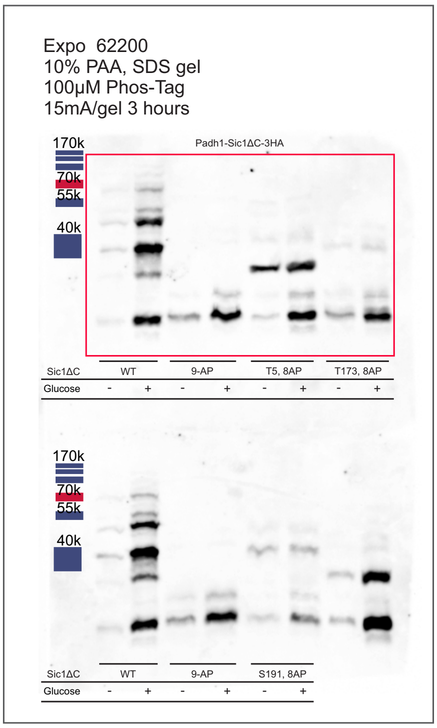

Figure 4d. Immunoblot luminescence

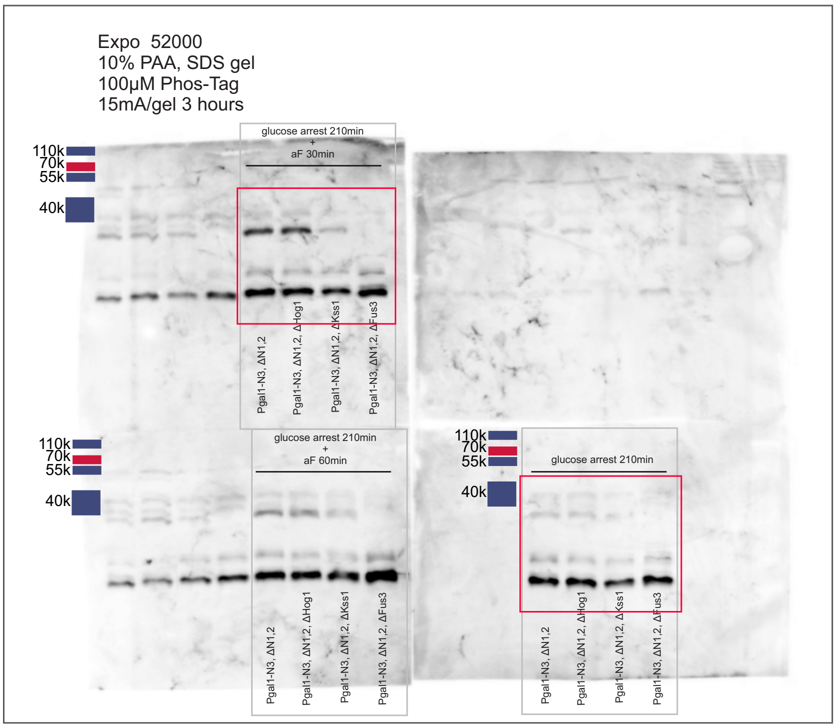

Supplementary Figure 7. Full scan images for Figure 4. For Figure 4a, c, d the uncropped images of Phos-Tag gel immunoblot luminescence images are presented. The proteins are detected by anti-HA tag (1:1000) and anti mouse-HRP (1:10,000) antibody complex and Image taken by ImageQuant RT ECL luminescence detector. For Figure 4b kinase assay SDS-PAGE 32P-autoradiograph is presented. The cropped areas for the figures are marked by the red line. Protein ladder used: PageRuler™ Prestained Protein Ladder, 10 to 180 kDa (Thermo Scientific, cat # 26617).

Figure 4e. Immunoblot luminescence

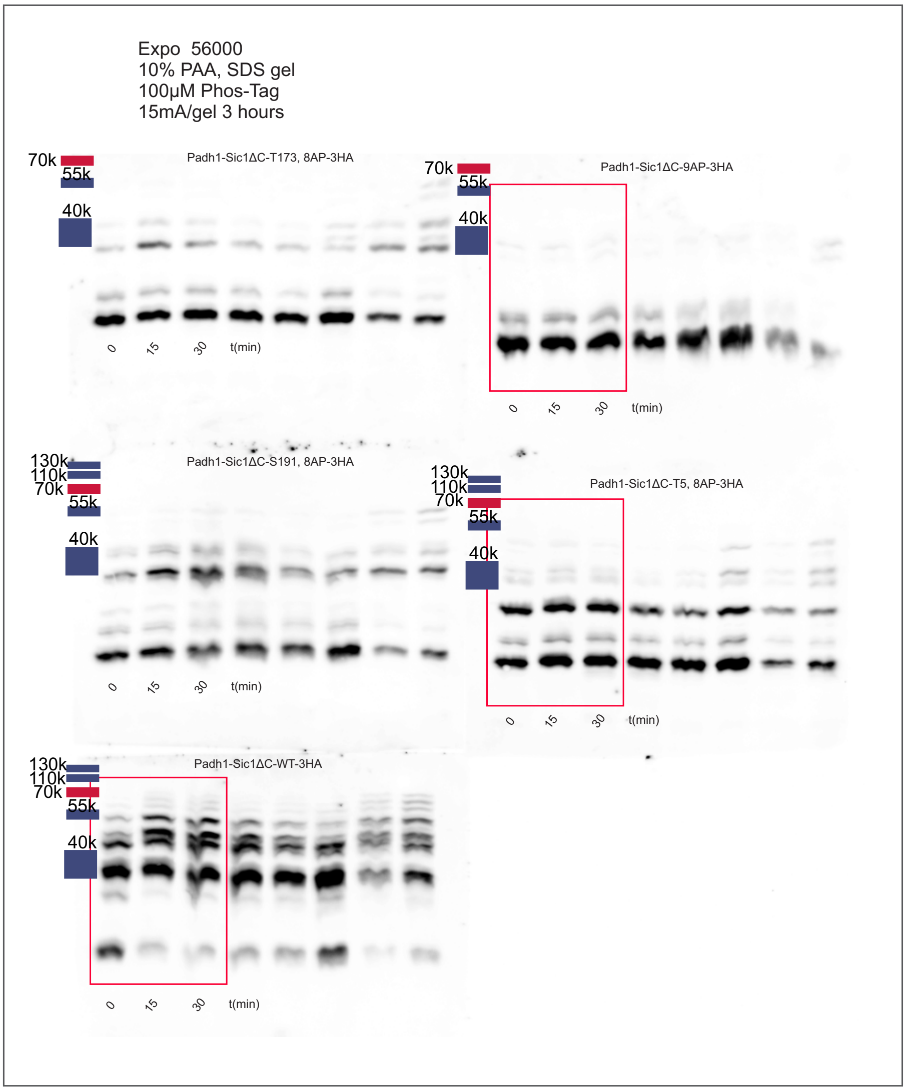

Supplementary Figure 7. Full scan images for Figure 4. For Figure 4e the uncropped image of Phos-Tag gel immunoblot luminescence image is presented. The proteins are detected by anti-HA tag (1:1000) and anti mouse-HRP (1:10,000) antibody complex and Image taken by ImageQuant RT ECL luminescence detector. The cropped areas for the figures are marked by the red line. Protein ladder used: PageRuler™ Prestained Protein Ladder, 10 to 180 kDa (Thermo Scientific, cat # 26617).

Supplementary Table 1. The single cell analysis of Sic1 destruction timing from Whi5 nuclear exit with 95% confidence interval (CI) values and numbers of individual cells (n) analyzed.

Growth conditions	Sic1	CDK inhibitory domain	Mother/ daughter/ all cells	Sic1 destruction timing			
				median value (min)	upper 95% CI	lower 95% CI	(n)
Unperturbed cells	WT	full length (aa 1-284)	All cells	14.2	15.3	13.5	112
			Mother	13.7	14.2	12.1	43
			Daughter	15.3	16.8	13.5	69
	T2,5S		All cells	17.6	19.2	16.0	90
			Mother	13.1	15.6	12.1	29
			Daughter	20.2	21.8	17.9	61
	VLLPP		All cells	18.4	20.2	16.9	98
			Mother	14.7	16.5	12.9	47
			Daughter	21.4	23.3	20.2	51
	T2,5S-VLLPP		All cells	31.8	34.9	29.4	118
			Mother	28.1	30.0	25.1	65
			Daughter	39.8	44.7	34.9	53
	T2,5S-VLLPP-T173S		All cells	32.5	34.1	30.0	110
			Mother	27.5	30.0	23.4	43
			Daughter	34.6	40.2	32.5	67
	RXL		All cells	17.7	18.9	17.1	161
			Mother	17.1	17.7	15.3	78
			Daughter	19.5	22.0	18.3	83
	T2,5S-RXL		All cells	26.2	27.5	24.4	98
			Mother	21.8	23.4	19.3	41
			Daughter	29.5	35.1	26.5	57
	RXL-VLLPP		All cells	24.7	26.9	23.4	98
			Mother	19.6	21.4	17.8	40
			Daughter	28.2	30.0	26.5	58
	T173S		All cells	14.1	15.6	13.1	84
			Mother	14.1	16.5	12.2	35
			Daughter	14.1	15.6	12.1	49
	9SP		All cells	89.5	92.6	83.4	107
			Mother	79.3	88.0	72.2	57
			Daughter	94.3	107.8	90.5	50
	SEE NEXT PAGE						
	WT	ΔC (aa 1-215)	All cells	18.7	19.9	16.7	70
			Mother	17.1	19.5	15.1	30
			Daughter	19.9	22.8	18.3	40
	T2,5S		All cells	17.6	18.4	17.1	267

			Mother	15.9	17.1	15.1	102
			Daughter	19.5	20.3	18.4	165
	VLLPP		All cells	17.1	18.7	15.4	91
			Mother	14.3	15.4	13.2	40
			Daughter	20.4	22.0	19.3	51
	T2,5S-VLLPP		All cells	16.3	17.1	15.8	249
			Mother	14.3	15.1	13.8	98
			Daughter	17.9	18.7	17.1	151
	RXL		All cells	25.1	27.5	23.4	86
			Mother	21.2	27.5	18.3	24
			Daughter	26.8	28.6	24.2	62
	T2,5S-RXL		All cells	24.2	25.6	22.8	141
			Mother	21.2	23.6	18.3	48
			Daughter	26.4	27.3	24.4	93
	T2,5S-RXL-VLLPP		All cells	31.3	33.0	30.0	178
			Mother	27.3	29.3	24.4	56
			Daughter	34.0	35.9	32.1	122
	T173S		All cells	18.9	20.3	17.9	70
			Mother	17.7	18.7	15.9	38
			Daughter	20.5	22.0	19.1	32
	9SP		All cells	50.9	53.7	48.4	102
			Mother	48.4	52.9	41.1	29
			Daughter	52.1	56.0	48.5	73
Pheromone-pulse intercepted cells	WT	full length (aa 1-284)	All cells	11.0	12.7	9.8	94
	T2,5S		All cells	19.3	23.4	15,3	45
	VLLPP		All cells	20.4	22.8	18,3	68
	T2,5S-VLLPP		All cells	37.1	43.2	29,0	41
	RXL		All cells	16.3	19.3	14,2	35
	T2,5S-RXL		All cells	25.0	30.5	21,3	27
	T173S		All cells	-24.3	-5.1	-43.9	34
	T173A		All cells	-15.4	-7.6	-26.1	52
	9SP		All cells	78.1	101.9	61.5	17
	T173-2P+3KA		All cells	13.2	26.8	9.7	11
	3XT5-NT-cluster		All cells	9.2	12.1	7.3	20
	WT	ΔC (aa 1-215)	All cells	12.2	15.9	8.5	21
	T173S		All cells	11.6	15.9	9.1	17

In 1% EtOH, 2% glycerol, unadapted to media	WT	full length (aa 1-284)	All cells	23.5	27.0	21.4	69
			Mother	21.4	24.2	18.5	29
			Daughter	26.0	29.9	23.5	40
	T173S		All cells	11.5	15.0	9.15	78
			Mother	10.0	17.8	0.20	17
			Daughter	12.8	16.4	9.20	61
In 1% EtOH, 2% glycerol, adapted to media	WT		All cells	20.4	21.4	18.9	166
			Mother	20.0	21.4	18.5	98
			Daughter	20.6	23.5	18.9	68
	T2,5S		All cells	28.5	30.6	27.8	247
			Mother	27.8	30.6	26.3	149
			Daughter	30.6	32.0	28.5	98
	T173S		All cells	15.8	17.7	12.8	94
			Mother	15.8	18.3	11.6	59
			Daughter	14.3	18.3	11.4	35
	T2,5S,T173S		All cells	22.8	24.2	21.3	125
			Mother	21.0	22.8	19.9	90
			Daughter	25.6	29.9	22.8	35

Supplementary Table 2. Yeast strains used in this study

Strain	Purpose / figure	Description
DOM0090	Parental strain	w303: MATA {*leu2-3,112 trp1-1 can1-100 ura3-1 ade2-1 his3-11,15 bar1::hisG}* [*phi+*]
DOM0030	Parental strain	DOM0090, *cdc28::cdc28(as1)*
DK252	Parental strain<br>Fig. 6e	w303: MATA, *cln1::HIS3, cln2::LEU2, ura3::*P_{GAL1}*-CLN3*
RV793	Fig. 4a	DOM0090, [pRS315-P_{ADH1}-WT-Sic1ΔC-3HA]
RV795	Fig. 4a	DOM0090, [pRS315-P_{ADH1}-9AP-Sic1ΔC-3HA]
RV797	Fig. 4a	DOM0090, [pRS315-P_{ADH1}-8AP-T2-Sic1ΔC-3HA]
RV799	Fig. 4a	DOM0090, [pRS315-P_{ADH1}-8AP-T5-Sic1ΔC-3HA]
RV801	Fig. 4a	DOM0090, [pRS315-P_{ADH1}-8AP-T33-Sic1ΔC-3HA]
RV803	Fig. 4a	DOM0090, [pRS315-P_{ADH1}-8AP-T45-Sic1ΔC-3HA]
RV805	Fig. 4a	DOM0090, [pRS315-P_{ADH1}-8AP-S69-Sic1ΔC-3HA]
RV807	Fig. 4a	DOM0090, [pRS315-P_{ADH1}-8AP-S76-Sic1ΔC-3HA]
RV809	Fig. 4a	DOM0090, [pRS315-P_{ADH1}-8AP-S80-Sic1ΔC-3HA]
RV811	Fig. 4a	DOM0090, [pRS315-P_{ADH1}-8AP-T173-Sic1ΔC-3HA]
RV813	Fig. 4a	DOM0090, [pRS315-P_{ADH1}-8AP-S191-Sic1ΔC-3HA]
RV282	Fig. 4c,e	DK252, [pRS316-P_{ADH1}-WT-Sic1ΔC-3HA]
RV283	Fig. 4c,e	DK252, [pRS316-P_{ADH1}-9AP-Sic1ΔC-3HA]
RV284	Fig. 4c,e	DK252, [pRS316-P_{ADH1}-8AP-T5-Sic1ΔC-3HA]
RV285	Fig. 4c,d	DK252, [pRS316-P_{ADH1}-8AP-T173-Sic1ΔC-3HA]
RV286	Fig. 4c	DK252, [pRS316-P_{ADH1}-8AP-S191-Sic1ΔC-3HA]
RV371	Fig. 4d	DK252, fus3::ura-x, [pRS316-P_{ADH1}-8AP-T173-Sic1ΔC-3HA]
RV373	Fig. 4d	DK252, hog1::ura-x, [pRS316-P_{ADH1}-8AP-T173-Sic1ΔC-3HA]
RV375	Fig. 4d	DK252, kss1::ura-x, [pRS316-P_{ADH1}-8AP-T173-Sic1ΔC-3HA]
RV200	Fig. 5b,d,e,g,j,k,m;<br>Fig. 6a,c;<br>Fig. S3b,e,f,g;<br>Fig. S4d,e,f,g,h,i,j;<br>Fig. S5a,b,c,d;<br>Fig. S6a,b	DOM0090, *Whi5:mCherry-SpHIS5, Sic1:GFP-kanMX6, bar1::HisG*
RV396	Fig. 5c,d,e,h;<br>Fig. 6c;<br>Fig. S3b;<br>Fig. S4e,f,i,j;<br>Fig. S5a,b,c,d;<br>Fig. S6a,b	DOM0090, *Whi5:mCherry-SpHIS5, T173S-Sic1:GFP-kanMX6, bar1::HisG*
RV544	Fig. 5e,i;<br>Fig. 6c;<br>Fig. S4g,h,j;<br>Fig. S5c	DOM0090, *Whi5:mCherry-SpHIS5, 9SP-Sic1:GFP-kanMX6, bar1::HisG*
MO352	Fig. 5f	DOM0090*, Whi5:mCherry-SpHIS5, Clb5:mCitrine-KanMX, bar1::HisG*
RV553	Fig. 5j;<br>Fig. 6a,c;<br>Fig. S3g;<br>Fig. S4g,i,j;<br>Fig. S5a,b,c	DOM0090, *Whi5:mCherry-SpHIS5, T2,5S-VLLPPmut-Sic1:GFP-kanMX6, bar1::HisG*

RV556	Fig. 5k;<br>Fig. 6a,c;<br>Fig. S4g,i,j;<br>Fig. S5a,b,c	DOM0090, *Whi5:mCherry-SpHIS5, AXA2,3-Sic1:GFP-kanMX6, bar1::HisG*
RV546	Fig. 5k	DOM0090, *Whi5:mCherry-SpHIS5, T173-2P+3KA-Sic1:GFP-kanMX6, bar1::HisG*
RV154	Fig. 5m,	DOM0090, *Whi5:mCherry-SpHIS5, 3XN-T5primer-Sic1:GFP-kanMX6, bar1::HisG*
RV543	Fig. 6a,c;<br>Fig. S3g;<br>Fig. S4g,i,j;<br>Fig. S5a,b,c	DOM0090, *Whi5:mCherry-SpHIS5, T2,5S-Sic1:GFP-kanMX6, bar1::HisG*
RV550	Fig. 6a,c;<br>Fig. S3g;<br>Fig. S4g,i,j;<br>Fig. S5a,b,c	DOM0090, *Whi5:mCherry-SpHIS5, VLLPPmut-Sic1:GFP-kanMX6, bar1::HisG*
RV600	Fig. 6a,c;<br>Fig. S5a,b,c	DOM0090, *Whi5:mCherry-SpHIS5, T2,5S,AXA2,3-Sic1:GFP-kanMX6, bar1::HisG*
RV949	Fig. 6b,c;<br>Fig. S5a,b,c	DOM0090, *Whi5:mCherry-SpHis5, bar1::HisG, 2X[StuI, pRS306-*P_{SIC1}*-WT-Sic1ΔC-wt-linker-GFP]*
RV951	Fig. 6c;<br>Fig. S5c	DOM0090, *Whi5:mCherry-SpHis5, bar1::HisG, 2X[StuI, pRS306-*P_{SIC1}*-T173S-Sic1ΔC-linker-GFP]*
RV1000	Fig. 6b,c;<br>Fig. S5a,b,c	DOM0090, *Whi5:mCherry-SpHis5, bar1::HisG, 1X[StuI, pRS306-*P_{SIC1}*-T2,5S-Sic1ΔC-linker-GFP]*
RV1008	Fig. 6b,c;<br>Fig. S5a,b,c	DOM0090, *Whi5:mCherry-SpHis5, bar1::HisG, 2X[StuI, pRS306-*P_{SIC1}*-VLLPPmut-Sic1ΔC-linker-GFP]*
RV953	Fig. 6b,c;<br>Fig. S5a,b,c	DOM0090, *Whi5:mCherry-SpHis5, bar1::HisG, 1X[StuI, pRS306-*P_{SIC1}*-T2,5S,VLLPPmut-Sic1ΔC-linker-GFP]*
RV955	Fig. 6b,c;<br>Fig. S5a,b,c	DOM0090, *Whi5:mCherry-SpHis5, bar1::HisG, 2X[StuI, pRS306-*P_{SIC1}*-AXA2,AXA3-Sic1ΔC-linker-GFP]*
RV1013	Fig. 6b,c;<br>Fig. S5a,b,c	DOM0090, *Whi5:mCherry-SpHis5, bar1::HisG, 1X[StuI, pRS306-*P_{SIC1}*-T2,5S,AXA2,AXA3-Sic1ΔC-linker-GFP]*
RV1011	Fig. 6b,c;<br>Fig. S5a,b,c	DOM0090, *Whi5:mCherry-SpHis5, bar1::HisG, 2X[StuI, pRS306-*P_{SIC1}*-T2,5S,VLLPPmut,AXA2,3-Sic1ΔC-linker-GFP]*
RV992	Fig. 6c;<br>Fig. S5c	DOM0090, Whi5:mCherry-SpHis5, bar1::HisG, 1X[StuI, pRS306-P_{SIC1}-9SP-Sic1ΔC-linker-GFP]
EV0013	Fig. 6d;<br>Fig. S3a;	DOM0090, *His3:*P_{GAL1}*-3HA-Clb5*
RV1119	Fig. 6d	DOM0090, *His3:*P_{GAL1}*-3HA-Clb5, Sic1::9SP-Sic1*
RV1123	Fig. 6d	DOM0090, *His3:*P_{GAL1}*-3HA-Clb5, Sic1:: -8SP-T173-Sic1*
RV931	Fig. 6d	DK252, *Sic1::URA3*
RV1137	Fig. 6d	DK252, *Sic1::T173A-Sic1*
RV703	Fig. S1f	DOM0090, *9AP-Sic1:6XHA:kanMX6, bar1::HisG*
RV704	Fig. S1f	DOM0090, *8AP-T173-Sic1:6XHA:kanMX6, bar1::HisG*
RV622	Fig. S2a	DOM0090, *WT-Sic1:6XHA:kanMX6, bar1::HisG*
RV777	Fig. S2a	DOM0090, *[pRS306-*P_{SIC1}*-WT-Sic1ΔC-6HA]*
RV277	Fig. S2a	DOM0090, [pRS316-P_{ADH1}-WT-Sic1ΔC-3HA]
RV1127	Fig. S2b	DOM0090, *cln3::URA3*, [pRS315-P_{ADH1}-WT-Sic1ΔC-3HA]
RV1128	Fig. S2b	DOM0090, *cln3::URA3*, [pRS315-P_{ADH1}- T173A,S191A-Sic1ΔC-3HA]
RV1129	Fig. S2b	DOM0090, *cln3::URA3*, [pRS315-P_{ADH1}-8AP-T5-Sic1ΔC-3HA]
RV1130	Fig. S2b	DOM0090, *cln3::URA3*, [pRS315-P_{ADH1}-8AP-T173-Sic1ΔC-3HA]
RV1131	Fig. S2b	DOM0090, *cln3::URA3*, [pRS315-P_{ADH1}-8AP-S191-Sic1ΔC-3HA]

RV1132	Fig. S2b	DOM0090, *cln3::URA3*, [pRS315-P_{ADH1}-7AP-T173,S191-Sic1ΔC-3HA]
RV912	Fig. S2c	DOM0030, [pRS316-P_{ADH1}-8AP-T5-Sic1ΔC-3HA]
RV913	Fig. S2c	DOM0030, [pRS316-P_{ADH1}-8AP-T173-Sic1ΔC-3HA]
RV911	Fig. S2c supporting	DOM0030, [pRS316-P_{ADH1}-9AP-Sic1ΔC-3HA]
RV914	Fig. S2c supporting	DOM0030, [pRS316-P_{ADH1}-8AP-S191-Sic1ΔC-3HA]
YKO0001[6]	Fig. S2d	BY4741: MATA, *his3Δ0, leu2Δ0, met15Δ0, ura3Δ0, BAR1+*
YKO0802[6]	Fig. S2d	BY4741: MATA, *his3Δ0, leu2Δ0, met15Δ0, ura3Δ0, BAR1+, Sky1::kanMX6*
YKO0993[6]	Fig. S2d	BY4741: MATA, *his3Δ0, leu2Δ0, met15Δ0, ura3Δ0, BAR1+, Slt2::kanMX6*
YKO1137[6]	Fig. S2d	BY4741: MATA, *his3Δ0, leu2Δ0, met15Δ0, ura3Δ0, BAR1+, Mck1::kanMX6*
YKO1317[6]	Fig. S2d	BY4741: MATA, *his3Δ0, leu2Δ0, met15Δ0, ura3Δ0, BAR1+, Ime2::kanMX6*
YKO1507[6]	Fig. S2d	BY4741: MATA, *his3Δ0, leu2Δ0, met15Δ0, ura3Δ0, BAR1+, Kns1::kanMX6*
YKO2786[6]	Fig. S2d	BY4741: MATA, *his3Δ0, leu2Δ0, met15Δ0, ura3Δ0, BAR1+, Ssn3::kanMX6*
YKO3776[6]	Fig. S2d	BY4741: MATA, *his3Δ0, leu2Δ0, met15Δ0, ura3Δ0, BAR1+, Mrk1::kanMX6*
YKO6278[6]	Fig. S2d	BY4741: MATA, *his3Δ0, leu2Δ0, met15Δ0, ura3Δ0, BAR1+, Ygk3::kanMX6*
YKO6745[6]	Fig. S2d	BY4741: MATA, *his3Δ0, leu2Δ0, met15Δ0, ura3Δ0, BAR1+, Rim11::kanMX6*
YKO6981[6]	Fig. S2d	BY4741: MATA, *his3Δ0, leu2Δ0, met15Δ0, ura3Δ0, BAR1+, Kss1::kanMX6*
YKO7006[6]	Fig. S2d	BY4741: MATA, *his3Δ0, leu2Δ0, met15Δ0, ura3Δ0, BAR1+, Yak1::kanMX6*
YKO1428[6]	Fig. S2d	BY4741: MATA, *his3Δ0, leu2Δ0, met15Δ0, ura3Δ0, BAR1+, Cka1::kanMX6*
YKO1837[6]	Fig. S2d	BY4741: MATA, *his3Δ0, leu2Δ0, met15Δ0, ura3Δ0, BAR1+, Cka2::kanMX6*
YKO5810[6]	Fig. S2d	BY4741: MATA, *his3Δ0, leu2Δ0, met15Δ0, ura3Δ0, BAR1+, Kin82::kanMX6*
YKO7028[6]	Fig. S2d	BY4741: MATA, *his3Δ0, leu2Δ0, met15Δ0, ura3Δ0, BAR1+, Ctk1::kanMX6*
RV589	Fig. S2d	YKO0020, *Pho85::ura-x*
YKO3042[6]	Fig. S2d	BY4741: MATA, *his3Δ0, leu2Δ0, met15Δ0, ura3Δ0, BAR1+, Fus3::kanMX6*
YKO5011[6]	Fig. S2d	BY4741: MATA, *his3Δ0, leu2Δ0, met15Δ0, ura3Δ0, BAR1+, Kdx1::kanMX6*
YKO2797[6]	Fig. S2d	BY4741: MATA, *his3Δ0, leu2Δ0, met15Δ0, ura3Δ0, BAR1+, Pho85::kanMX6*
YKO1910[6]	Fig. S2d	BY4741: MATA, *his3Δ0, leu2Δ0, met15Δ0, ura3Δ0, BAR1+, Ksp1::kanMX6*
YKO5473[6]	Fig. S2d	BY4741: MATA, *his3Δ0, leu2Δ0, met15Δ0, ura3Δ0, BAR1+, Smk1::kanMX6*
YKO2724[6]	Fig. S2d	BY4741: MATA, *his3Δ0, leu2Δ0, met15Δ0, ura3Δ0, BAR1+, Hog1::kanMX6*
YKO0415[6]	Fig. S2d	BY4741: MATA, *his3Δ0, leu2Δ0, met15Δ0, ura3Δ0, BAR1+, Kin3::kanMX6*
YKO2707[6]	Fig. S2d	BY4741: MATA, *his3Δ0, leu2Δ0, met15Δ0, ura3Δ0, BAR1+, Kin2::kanMX6*
YKO2058[6]	Fig. S2d	BY4741: MATA, *his3Δ0, leu2Δ0, met15Δ0, ura3Δ0, BAR1+, Yck2::kanMX6*
YKO3798[6]	Fig. S2d	BY4741: MATA, *his3Δ0, leu2Δ0, met15Δ0, ura3Δ0, BAR1+, Dun1::kanMX6*
YKO5271[6]	Fig. S2d	BY4741: MATA, *his3Δ0, leu2Δ0, met15Δ0, ura3Δ0, BAR1+, Ste11::kanMX6*
YKO5021[6]	Fig. S2d	BY4741: MATA, *his3Δ0, leu2Δ0, met15Δ0, ura3Δ0, BAR1+, Nnk1::kanMX6*
YKO5722[6]	Fig. S2d	BY4741: MATA, *his3Δ0, leu2Δ0, met15Δ0, ura3Δ0, BAR1+, Chk1::kanMX6*
YKO6589[6]	Fig. S2d	BY4741: MATA, *his3Δ0, leu2Δ0, met15Δ0, ura3Δ0, BAR1+, Bub1::kanMX6*
YKO4389[6]	Fig. S2d	BY4741: MATA, *his3Δ0, leu2Δ0, met15Δ0, ura3Δ0, BAR1+, Alk1::kanMX6*
YKO6128[6]	Fig. S2d	BY4741: MATA, *his3Δ0, leu2Δ0, met15Δ0, ura3Δ0, BAR1+, Sak1::kanMX6*
YKO7552[6]	Fig. S2d	BY4741: MATA, *his3Δ0, leu2Δ0, met15Δ0, ura3Δ0, BAR1+, Abc1::kanMX6*
YKO0878[6]	Fig. S2d	BY4741: MATA, *his3Δ0, leu2Δ0, met15Δ0, ura3Δ0, BAR1+, Mec1::kanMX6*
YKO1238[6]	Fig. S2d	BY4741: MATA, *his3Δ0, leu2Δ0, met15Δ0, ura3Δ0, BAR1+, Swe1::kanMX6*
YKO1907[6]	Fig. S2d	BY4741: MATA, *his3Δ0, leu2Δ0, met15Δ0, ura3Δ0, BAR1+, Ire1::kanMX6*
YKO4341[6]	Fig. S2d	BY4741: MATA, *his3Δ0, leu2Δ0, met15Δ0, ura3Δ0, BAR1+, Gin4::kanMX6*
YKO5016[6]	Fig. S2d	BY4741: MATA, *his3Δ0, leu2Δ0, met15Δ0, ura3Δ0, BAR1+, Tpk3::kanMX6*

EV0026	Fig. S3a	DOM0090, *His3:*P_{GAL1}*-3HA-Clb5, Sic1::T173A-Sic1*
RV218	Fig. S3e	DOM0090, *Whi5:mCherry-SpHIS5, T173A-Sic1:GFP-kanMX6, bar1::HisG*
RV982	Fig. S3f;<br>Fig. S6a,b	DOM0090, *Whi5:mCherry-SpHis5, bar1::HisG, 1X[StuI, pRS306-*P_{sic1}*-WT-Sic1ΔC-linker-GFP]*
RV978	Fig. S3f;<br>Fig. S6a,b	DOM0090, *Whi5:mCherry-SpHis5, bar1::HisG, 1X[StuI, pRS306-*P_{SIC1}*-T173S-Sic1ΔC-linker-GFP]*

Supplementary Table 3. Plasmids used in this study

Plasmid	Description	Plasmid	Description
PSIC1	pET28a(+)-6xHis-WT-Sic1	PSIC26	pET28a(+)-6xHis-8AP-S191-Sic1
PSIC2	pET28a(+)-6xHis-Sic1ΔC	PSIC112	pET28a(+)-6xHis- AXA2,3-Sic1
PSIC3	pET28a(+)-6xHis-T5A-Sic1	PSIC114	pET28a(+)-6xHis- AXA5-Sic1
PSIC108	pET28a(+)-6xHis-T2,5S-Sic1	PSIC83	pRS306-P_{SIC1}-WT-Sic1ΔC-6xHA
PSIC4	pET28a(+)-6xHis-T33A-Sic1	PSIC85	pRS306-P_{SIC1}-9AP-Sic1ΔC-6xHA
PSIC5	pET28a(+)-6xHis-T45A-Sic1	PSIC87	pRS306-P_{SIC1}-8AP-T173-Sic1ΔC-6xHA
PSIC6	pET28a(+)-6xHis-T48A-Sic1	PSIC89	pRS306-P_{SIC1}-8AP-T5-Sic1ΔC-6xHA
PSIC7	pET28a(+)-6xHis-S69A-Sic1	PSIC91	pRS316-P_{ADH1}-WT-Sic1ΔC-3xHA
PSIC8	pET28a(+)-6xHis-S76A-Sic1	PSIC93	pRS316-P_{ADH1}-9AP-Sic1ΔC-3xHA
PSIC9	pET28a(+)-6xHis-S80A-Sic1	PSIC95	pRS316-P_{ADH1}-8AP-T5-Sic1ΔC-3xHA
PSIC10	pET28a(+)-6xHis-T173A-Sic1	PSIC97	pRS316-P_{ADH1}-8AP-T173-Sic1ΔC-3xHA
PSIC110	pET28a(+)-6xHis-T173S-Sic1	PSIC99	pRS316-P_{ADH1}-8AP-S191-Sic1ΔC-3xHA
PSIC11	pET28a(+)-6xHis-S191A-Sic1	PSIC101	pRS316-P_{ADH1}-7AP-T173, S191-Sic1ΔC-3xHA
PSIC12	pET28a(+)-6xHis-T173A,S191A-Sic1	PSIC36	pRS306-P_{SIC1}-WT-Sic1ΔC-GFP
PSIC103	pET28a(+)-6xHis-T2,5S,T173S-Sic1	PSIC38	pRS306-P_{SIC1}-T173S-Sic1ΔC-GFP
PSIC13	pET28a(+)-6xHis-8AP-T2-Sic1	PSIC40	pRS306-P_{SIC1}-T2,5S,VLLPPmut-Sic1ΔC-GFP
PSIC15	pET28a(+)-6xHis-8AP-T33-Sic1	PSIC42	pRS306-P_{SIC1}-AXA2,3-Sic1ΔC-GFP
PSIC17	pET28a(+)-6xHis-8AP-T45-Sic1	PSIC65	pRS306-P_{SIC1}-9SP-Sic1ΔC-GFP
PSIC18	pET28a(+)-6xHis-8AP-T5-Sic1	PSIC67	pRS306-P_{SIC1}-8SP-T173-Sic1ΔC-GFP
PSIC20	pET28a(+)-6xHis-9AP-Sic1	PSIC69	pRS306-P_{SIC1}-T2,5S-Sic1ΔC-GFP
PSIC34	pET28a(+)-6xHis-9SP-Sic1	PSIC75	pRS306-P_{SIC1}-VLLPPmut-Sic1ΔC-GFP
PSIC21	pET28a(+)-6xHis-8AP-S69-Sic1	PSIC76	pRS306-P_{SIC1}-AXA2,3,VLLPPmut-Sic1ΔC-GFP
PSIC22	pET28a(+)-6xHis-8AP-S76-Sic1	PSIC77	pRS306-P_{SIC1}-T2,5S,AXA2,3,VLLPPmut-Sic1ΔC-GFP
PSIC23	pET28a(+)-6xHis-8AP-S80-Sic1	PSIC79	pRS306-P_{SIC1}-T2,5S,AXA2,3-Sic1ΔC-GFP
PSIC24	pET28a(+)-6xHis-8AP-T173-Sic1	PSIC116	pRS426-P_{GAL1}-Clb5-TAP

Supplementary Table 4. Primers used in this study

Number/primer name	Sequence
38/Sic1-rev-BamHI	ggttgcactggacagggatcctcaatgctcttgatccctag
242/Sic1-fwr-AP-NheI	gacagcgctagcatggccccttccgccccaccaaggtccagag
321/Sic1-fwr-2AP-Nhel	gacagcgctagcatggccccc
572/Sic1-del-URA-F	acggaattttgacccttgaagcagggactattacacgaaacgtttcggttgatgac
575/Sic1-wt-back-F	acggaattttgacccttgaagcagggactattacacgaaaatgactccttccacccca
625/Sic1-5AP-back-F	ttttgacccttgaagcagggactattacacgaaaatgactccttccgcccc
627/Sic1-wt-Nhel-F	cagcgctagcatgactccttccaccccaccaaggtccagag
665/Sic1-fwr-2/5AP-BamHI	gacagcggatccatggccccttccgccccaccaaggtccagaggg
667/Sic1-fwr-2AP-Nhel	gacagcgctagcatggccccctccaccccaccaaggtccagaggg
678/Sic1-fwr-5AP-Nhel	gacagcgctagcatgactccttccgccccaccaaggtccagaggg
711/Sic1-3HA-Ncol-R	gcccatggtcaagcgtaatctggaacatcgtatgggtaagcgtaatctggaacatcgtatgggtaagcgtaatctgga acatcgtatgggtaatgctcttgatccctag
750/Sic1-3HA-cdkXbalR	ccgctctagatcaagcgtaatctggaacatcgtatgggtaagcgtaatctggaacatcgtatgggtaagcgtaatctgg aacatcgtatgggtaacccacccgcactgg
865/Sic1-p-HindIII-F	gcagaagcttgaattcttttcaaacctgg
866/Sic1-p-Smal-R	ctgccccgggtttcgtgtaatagtccc
1546/Sic1-cdkinh-BamHI	gcactggacagggatcctcaacccacccgcactgg
1602/Sic1-2S/5S-Nhel-F	gcatttgctagcatgagcccttccagcccaccaaggtccagaggg
1796/NSic1_del_R	ttaaccttcgtttctctaccacctctcccttcttattgacttcctgatgcggtattttctcct
1797/NSic1_back_R	ttaaccttcgtttctctaccacc
2067/Sic1 -RT-F	tcagatgaactggtcactcagg
2068/Sic1 -RT-R	ctcttttcttcatccgttaacc
2180/Sic1-Ser-back-F	acggaattttgacccttgaagcagggactattacacgaaaatgagcccttccagcccaccaaagtccag
2231/Sic1dC-EGFP-F	tcaatccagtgcgggtgggtatccccgggttaattaaca
2232/Sic1dC-EGFP-R	tgttaattaacccggggatacccacccgcactggattga
2233/EGFP_XbaI_R	aaaatctagatcataaatcataagaaattc
2309/Sic1-p-BamHI-R	tatataggatcctttcgtgtaatagtccct

*In this table only unique oligos related to Sic1 are listed. For extended list including all oligonucleotide sequences available used for oligo-directed mutagenesis or standard use of genomic deletion, tagging and sequencing will be made available upon request from corresponding author Mart Loog (mart.loog@ut.ee).

Supplementary references

1. Edgar, R.C. MUSCLE: multiple sequence alignment with high accuracy and high throughput. Nucleic Acids Res. **32**, 1792–1797 (2004).
2. Koivomagi, M. *et al.* Multisite phosphorylation networks as signal processors for Cdk1. *Nat. Struct. Mol. Biol.* **20**, 1415–1424 (2013).
3. Koivomagi, M. *et al.* Cascades of multisite phosphorylation control Sic1 destruction at the onset of S phase. *Nature* **480**, 128–131 (2011).
4. Bhaduri, S., Pryciak P.M. Cyclin-specific docking motifs promote phosphorylation of yeast signaling proteins by G1/S Cdk complexes. Curr Biol. **21**, 1615-23 (2011).
5. Schwob E., Nasmyth K. CLB5 and CLB6, a new pair of B cyclins involved in DNA replication in Saccharomyces cerevisiae. Genes Dev. **7**, 1160-75 (1993).
6. Giaever, G. *et al.* Functional profiling of the Saccharomyces cerevisiae genome. *Nature* **418**, 387–391 (2002).
