# Supplementary material for: A processive phosphorylation circuit with multiple kinase inputs and mutually diversional routes controls G1/S decision
Source: Nat Commun. 2020 Apr 15;11:1836. doi: 10.1038/s41467-020-15685-z (PMC7160111; doi:10.1038/s41467-020-15685-z)
Supplement: Supplementary file 2 — Reporting Summary [file 41467_2020_15685_MOESM2_ESM.pdf]

nature research

Corresponding author(s): Mart Loog

Last updated by author(s): Mar 13, 2020

Reporting Summary

Nature Research wishes to improve the reproducibility of the work that we publish. This form provides structure for consistency and transparency in reporting. For further information on Nature Research policies, see Authors & Referees and the Editorial Policy Checklist.

Statistics

For all statistical analyses, confirm that the following items are present in the figure legend, table legend, main text, or Methods section.

n/a	Confirmed	
☐	☒	The exact sample size (*n*) for each experimental group/condition, given as a discrete number and unit of measurement
☐	☒	A statement on whether measurements were taken from distinct samples or whether the same sample was measured repeatedly
☒	☐	The statistical test(s) used AND whether they are one- or two-sided<br>*Only common tests should be described solely by name; describe more complex techniques in the Methods section.*
☒	☐	A description of all covariates tested
☒	☐	A description of any assumptions or corrections, such as tests of normality and adjustment for multiple comparisons
☐	☒	A full description of the statistical parameters including central tendency (e.g. means) or other basic estimates (e.g. regression coefficient) AND variation (e.g. standard deviation) or associated estimates of uncertainty (e.g. confidence intervals)
☒	☐	For null hypothesis testing, the test statistic (e.g. *F*, *t*, *r*) with confidence intervals, effect sizes, degrees of freedom and *P* value noted<br>*Give P values as exact values whenever suitable.*
☒	☐	For Bayesian analysis, information on the choice of priors and Markov chain Monte Carlo settings
☒	☐	For hierarchical and complex designs, identification of the appropriate level for tests and full reporting of outcomes
☒	☐	Estimates of effect sizes (e.g. Cohen's *d*, Pearson's *r*), indicating how they were calculated

Our web collection on statistics for biologists contains articles on many of the points above.

Software and code

Policy information about availability of computer code

Data collection	Live-cell microscopy data was collected using ZEN software (Zeiss, version 2.3, blue edition). The 32-P autoradiography images from kinase assays were quantified by using ImageQuant TL software (Amersham, version 8.1), as described in methods section.
Data analysis	Microscopy data was analyzed by using custom made scripts for image segmentation, cell tracking and quantification MATLAB (Mathworks, version R2016a). The scripts are available upon request.

For manuscripts utilizing custom algorithms or software that are central to the research but not yet described in published literature, software must be made available to editors/reviewers. We strongly encourage code deposition in a community repository (e.g. GitHub). See the Nature Research guidelines for submitting code & software for further information.

Data

Policy information about availability of data

All manuscripts must include a data availability statement. This statement should provide the following information, where applicable:

- Accession codes, unique identifiers, or web links for publicly available datasets
- A list of figures that have associated raw data
- A description of any restrictions on data availability

All data will be made available upon request from corresponding author Mart Loog (mart.loog@ut.ee).

Field-specific reporting

Please select the one below that is the best fit for your research. If you are not sure, read the appropriate sections before making your selection.

☒ Life sciences ☐ Behavioural & social sciences ☐ Ecological, evolutionary & environmental sciences

Life sciences study design

All studies must disclose on these points even when the disclosure is negative.

Sample size	No sample-size pre-calculations were made. For kinase assays, all experiments were repeated independently at least twice due to very low variability between the experiments. The low (<10%) variability was further corroborated in reactions with WT-Sic1 where the number of independent experiments performed was in some cases as high as 6. Therefore, and due to highly defined biochemical composition and defined reaction conditions, we were sufficiently satisfied with two independent experiments. In addition, all independent kinase assays were processed in two or more timepoints, which were not regarded as independent replicas, but further demonstrated very low technical variability of the assay. For in vivo experiments where the readout was processed in form of immunological detection (Western blot), all experiments were carried out at least twice, including preparing and processing cell cultures, applying experimental conditions for the cultures, recovering samples and processing them until the final signal detection and quantification.<br>In live-cell microscopy experiments with unperturbed cell cycle 50 cells from two distinct individual colonies were considered to be sufficient for the analysis, as the median values and their 95% confidence intervals showed very little variation (<10%) between different individual colonies of the same strain, and combined 95% confidence interval values were reasonably narrow. For mating pheromone intercepted live-cell microscopy experiments 15 individual cells from at least 5 distinct experiments with independent colonies were considered to be sufficient for the analysis, if obtained 95% confidence interval values were reasonably narrow. In some cases more cells were analyzed if more experiemntal data was available.
Data exclusions	No data was excluded from the analysis.
Replication	All data presented is from at least two replicate experiments, all replication events were successful and are included in the data.
Randomization	Samples were only grouped by yeast strains, no further allocations were made.
Blinding	Investigators were not blinded to the group allocation, however, automated analysis was used whenever possible.

Reporting for specific materials, systems and methods

We require information from authors about some types of materials, experimental systems and methods used in many studies. Here, indicate whether each material, system or method listed is relevant to your study. If you are not sure if a list item applies to your research, read the appropriate section before selecting a response.

Materials & experimental systems

n/a	Involved in the study
☐	☒ Antibodies
☒	☐ Eukaryotic cell lines
☒	☐ Palaeontology
☒	☐ Animals and other organisms
☒	☐ Human research participants
☒	☐ Clinical data

Methods

n/a	Involved in the study
☒	☐ ChIP-seq
☒	☐ Flow cytometry
☒	☐ MRI-based neuroimaging

Antibodies

Antibodies used	For detecting HA epitope the blotting was carried out by using anti-HA (mouse HA.11 clone 16B12 from Covance, cat# MMS-101R 1:1000 dilution) and anti-mouse-IgG HRP conjugated antibody (Labas AS, custom made, 1:0000 dilution). For detecting Cdk1 anti-Cdk1 antibody (goat anti-Cdc28 antibody yC-20, from SantaCruz Biotechnology, cat# sc-6709, 1:1000 dilution) and anti-goat-IgG HRP conjugated antibody (Labas AS, custom made, 1:0000 dilution) were used.<br>For detecting Cks1 anti-Cks1 (rabbit antibody from Labas AS, custom made, 1:1000 dilution) and anti-rabbit-IgG HRP conjugated antibodies (Labas AS, custom made, 1:10000 dilution) were used.
Validation	The anti-HA antibody from Covance was raised against the twelve amino acid peptide CYPYDVPDYASL in mous and veryfied by Wester blotting against Hemagglutinin tag. The anti-Cks1 antibody from Labas AS was raised against the full Cks1 protein in rabbit and verified by western blotting against the bacterially expressed and purified Cks1. The anti-Cdk1 antibody (Cdc28 Antibody, yC-20) from SantaCruz Biotechnology was raised against the C-terminal region of yeast Cdk1 protein in goat and verified by western blotting against the yeast Cdk1 protein.
